# Direct profiling of non-adenosines in poly(A) tails of endogenous and therapeutic mRNAs with Ninetails

**DOI:** 10.1038/s41467-025-57787-6

**Published:** 2025-03-18

**Authors:** Natalia Gumińska, Katarzyna Matylla-Kulińska, Paweł S. Krawczyk, Michał Maj, Wiktoria Orzeł, Zuzanna Mackiewicz, Aleksandra Brouze, Seweryn Mroczek, Andrzej Dziembowski

**Affiliations:** 1 Laboratory of RNA Biology, International Institute of Molecular and Cell Biology, Warsaw, Poland; 2 Faculty of Biology, University of Warsaw, Warsaw, Poland; 3 NatWest Group, Warsaw, Poland

**Keywords:** Machine learning, RNA quality control, Software, RNA vaccines, High-throughput screening

## Abstract

Stability and translation of mRNAs, both endogenous and therapeutic, is determined by poly(A) tail. Direct RNA sequencing enables single-molecule measurements of poly(A) lengths, avoiding amplification bias. It also holds potential for observation of non-adenosines within poly(A), known to influence mRNA fate. However, there is no computational method to detect composite tails in Direct Sequencing data. To address this gap, we introduce the Ninetails, a neural network-based tool that accurately identifies and quantifies non-adenosines in poly(A) tails. Examination of different biological contexts revealed widespread non-adenosine decorations, with frequencies influenced by the origin of poly(A) tails differing by mRNA class, cell type, and species. Notably, substrates of cytoplasmic TENT5-polymerases and mitochondrially encoded mRNAs are enriched in composite tails. For mRNA therapeutics, we show that the composition of poly(A) tails in mRNA vaccines is dynamic during its cellular lifetime and that the manufacturing protocol of synthetic mRNAs affects the purity of poly(A) tails.

## Abstract (web summary)

Ninetails, a neural network that uses Nanopore Direct RNA sequencing data to identify non-A residues in mRNA poly(A) tails is presented. Particularly non-A of therapeutic, mitochondrial, and TENT5A/C substrate mRNAs are highlighted in the paper.

## Introduction

Virtually all eukaryotic mRNAs are provided with a non-templated poly(A) tail. This appendage plays an essential role in determining mRNA fate through a direct link to export, translation initiation, and turnover^1–4^. Depending on the molecular context, the poly(A) tail can be the hallmark of maturation, enhance mRNA stability, or trigger its degradation^1,5^. A variety of enzymes are responsible for mRNA tailing^5–7^. In the nucleus, canonical poly(A) polymerases (PAPs) synthesize poly(A) tails in a fashion that is coupled to transcription termination^5^. Numerous members of the non-canonical terminal nucleotidyltransferase (TENT) superfamily can also produce poly(A) tails, mostly outside the nucleus^5,6^. In mitochondria, mtPAP (also known as TENT6) adds poly(A) tails de novo after endoribonucleolytic cleavage of a polycistronic precursor, while in the cytoplasm TENTs act by elongating pre-existing mRNA tails^3,5,6,8^.

Traditionally, poly(A) tails were considered homogeneous adenosine stretches. However, their nucleotide composition has been shown to be more diverse than previously thought. According to recent studies, various enzymes can post-transcriptionally decorate poly(A) tails with non-adenosines, which affects mRNA stability^2,4,5,9,10^. For instance, uridylation of the 3’-end of the poly(A) tail by TUT4 (TENT3A) and TUT7 (TENT3B) promotes rapid mRNA decay. In contrast, guanosines intervening adenosines incorporated by TENT4A/B are thought to prevent poly(A) trimming^4,9–13^. It has also been reported that non-adenosine nucleotides (cytidines, guanosines, and uridines) are frequently widespread along the entire span of the tail^2,9,10^. Nevertheless, it is not entirely clear what functional implications might result from the different nucleotide composition of the poly(A) tails. Many questions remain about the poly(A) tail complexity of endogenous transcripts and even more about synthetic therapeutic mRNAs.

As a consequence of the global pandemic, a promising field of mRNA vaccines has emerged and continues to grow. Two mRNA vaccines, BNT162b2 and mRNA-1273, commonly used to fight COVID-19, are produced in vitro by bacteriophage T7 RNA polymerase (T7 RNAP) on a DNA template. These molecules have a similar structure to typical eukaryotic mRNA, as they are equipped with a cap and a poly(A) tail^14–16^. Interestingly, both mRNA vaccines have distinct composite tails^16,17^. In BNT162b2, 30 initial adenosines are followed by 10 non-adenosines and another 70 adenosines^17^. In contrast, the mRNA-1273 tail contains approximately 100 adenosines and a terminal mΨCmΨAG pentamer, which is most likely the remnant of a restriction cleavage of the DNA template^16^. However, knowledge about the metabolism of poly(A) tails of therapeutic mRNAs remains sparse^16^. It is conceivable that a better understanding of the molecular consequences of the composite content of poly(A) tails could pave the way for more efficient and stable therapeutic mRNAs.

Despite their role in gene regulation, poly(A) tails were initially not feasible to study due to challenges posed by homopolymeric sequences^2,4,11,18^. Nevertheless, the poly(A)-landscape has attracted growing interest in recent years, leading to the development of new techniques^2,4,9,11,18,19^. There are several high-throughput methods for detecting and profiling non-adenosines in poly(A) tails, including TAIL-seq^11^, full-length mRNA sequencing (FLAM-seq^2^) and poly(A) inclusive RNA isoform sequencing (PAIso-seq^9^). Although these methods relying on next- and third-generation sequencing significantly advanced the understanding of the poly(A) repertoire, they have serious shortcomings. Their most important drawback is the amplification step. Both reverse transcription and polymerase chain reactions lead to undesirable variations in sequencing results^20–22^. Furthermore, the data obtained through the synthetic proxy rather than by the molecule of interest are inconsistent with respect to the prevalence of cytidine or guanosine^2,9,10^. Therefore, there is a clear need for the development of more reliable methods.

Direct RNA sequencing (DRS) from Oxford Nanopore Technologies (ONT) eliminates potential sources of amplification bias^21,23,24^. This platform enables accurate characterization of native RNA molecules and provides full-length, strand-specific reads. It relies on monitoring changes in electrical current when the molecule of interest passes through a protein pore. The resulting signal (squiggle) is then decoded into the underlying sequence by dedicated algorithms^4,18,21,24–26^.

Multiple reports demonstrate the successful use of DRS in poly(A) profiling^3,10,16,18,19,27–34^. In principle, it also has the potential to investigate the nucleotide composition of nascent poly(A) tails. However, no suitable bioinformatic tools have been published so far. Therefore, we decided to address this issue by developing Ninetails – an R package that leverages artificial intelligence to accurately discriminate characteristic current intensity data (signatures) corresponding to specific non-adenosines. Our work provides an efficient, high-throughput, and reproducible framework for the detection and quantification of non-adenosines in poly(A) tails based on DRS (Supplementary Fig. 1). Initial Ninetails applications in selected biological contexts revealed complex dynamics of both the lengths and composition of Moderna mRNA-1273 vaccine poly(A) tails in target cells and non-adenosine prevalence in cytoplasmic substrates of TENT5 poly(A) polymerases, as well as mitochondrial mRNA polyadenylated by mtPAP. We anticipate that Ninetails will advance the study of the biological functions of mixed tails in various model systems, facilitating the unlocking of the regulatory potential of poly(A) tails and contributing to the development of future generations of mRNA therapeutics.

## Results

### Convolutional neural network accurately detects and classifies non-adenosines

**Fig. 1:**
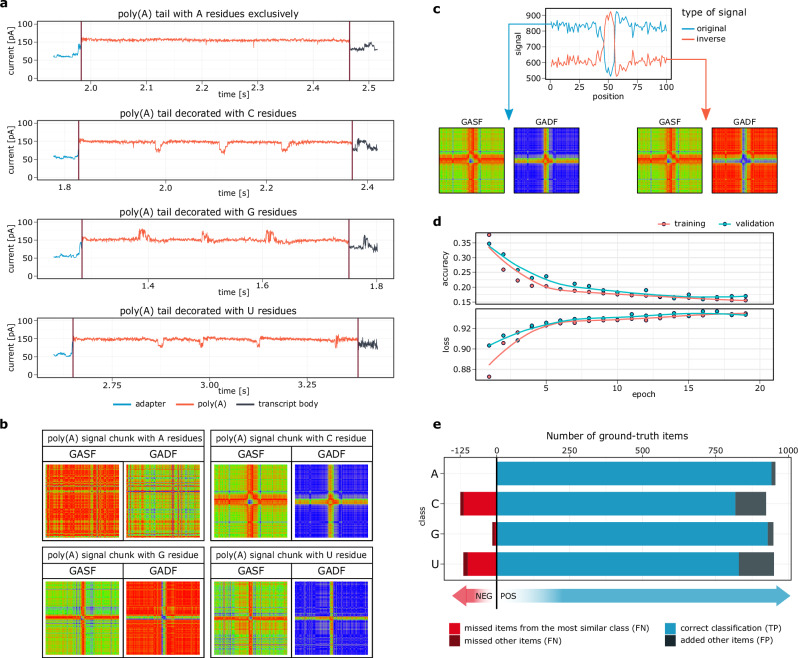
Convolutional Neural Network implemented in Ninetails enables precise detection of non-adenosines in Direct RNA Sequencing nanopore data. **a** Examples of DRS signals of in vitro transcribed molecules with blank tails (containing only adenosines) are compared with decorated tails in which cytidine, guanosine or uridine were inserted every 15 nucleotides, respectively. Maroon vertical lines denote poly(A) tail boundaries. **b** Example visualizations of the angular transformed nanopore signals. **c** A rationale for the applied signal transformation strategy. Signals that mirror each other (e.g. G and C/U) produce identical GASFs but different GADFs. **d** Training history for the applied CNN. **e** Visualization of the confusion matrix on the validation set. The color code distinguishes the items (signals) belonging to the most similar classes from the rest of the items. Predicted negatives (i.e. FN false negatives) are on the left side of the plot (red and maroon) while predicted positives (i.e. TP true positives and FP false positives) are on the right side of the plot (blue and steel).

The homopolymer stretches in DRS cannot be precisely basecalled due to the uniformity of the raw signal between adjacent nucleotides^18,19,35,36^. Nevertheless, we have found that non-adenosines in the poly(A) region alter the ionic current to such an extent that it can even be observed with the naked eye (Fig. 1a). Despite this, human vision fails to distinguish highly similar patterns, such as signatures of cytidines and uridines (Fig. 1a; Supplementary Fig. 2). However, this can be overcome by neural network-based algorithms, which have been proven to be highly successful with DRS data^25,26,35–40^. Therefore, we used this reasoning to develop our software (Supplementary Fig. 1).

To train and validate our model, we designed an experimental set of synthetic mRNA molecules with a uniform tail length (60 nucleotides) that either contain only adenosines or are decorated with a single cytidine, guanosine, or uridine in the middle of the homopolymer (Supplementary Figs. 1, 2b).

Ninetails extracts poly(A) tail signal and shifts in raw data representing potential nucleotide translocations (moves) from the basecalled sequencing files. Tail boundaries are determined according to the coordinates provided by the external software. By default, Ninetails accepts the predictions of nanopolish – software, which uses a predictive model combining a Hidden Markov Model (HMM) and an estimator of translocation rate through the pore^18^. However, it is also possible to use the outputs of tailfindR, which is delimiting poly(A) boundaries based on the slope of the raw signal, and normalization by the read-specific nucleotide translocation rate^19^ (Supplementary Notes 1, 2 and Supplementary Fig. 3). The raw signal is first interpolated and winsorized to eliminate sequencing artifacts and reduce background noise (Supplementary Note 3, Supplementary Fig. 4). Signal regions exhibiting a significant deviation from the mean are identified as potentially containing non-adenosines based on the two factors: moves (from the Guppy basecaller) and “pseudomoves” provided by the z-score thresholding algorithm^41^ (Supplementary Note 4, Supplementary Fig. 5). These fragments are extracted from the preprocessed signal with a fixed-length context centered on the signal anomaly. They are then encoded as the Gramian Angular Fields (GAFs), to expose features and patterns that cannot be found in the one-dimensional sequence of the original data^25,42^. We have implemented composite arrays of both Gramian Angular Summation Field (GASF) and Gramian Angular Difference Field (GADF) transformations (Fig. 1b; Supplementary Figs. 1, 2a, b). This prevents the scenario where two signals with point-wise opposite signs produce identical images, which affects the classification result. We found that this is the case for guanosines versus cytidines and uridines (Fig. 1c).

The resulting GADF/GASF dataset was used as input for the convolutional neural network (CNN) (Supplementary Fig. 1; for details see Materials and methods). We tested a variety of architectures, starting with the ResNet-20 recommended by Smith et al.^25^. Unfortunately, this proved too elaborate to yield satisfactory results despite model fine-tuning. To accommodate a relatively small input size, the CNN architecture had to be adjusted to avoid overtraining and a resulting decrease in prediction accuracy. Therefore, we built a much simpler model based on the principles of Visual Geometry Group (VGG) networks (Supplementary Data 1), which are known for remarkably satisfactory performance^43^.

Our best model achieved an overall accuracy and sensitivity (recall) of more than 93% and a specificity of ∼98%. The values of all calculated quality metrics demonstrate the high reliability of the model and the low probability of misclassification^44^. Detailed statistics for each class can be found in Supplementary Data 1. Receiver operating characteristic (ROC) analysis revealed an area under the curve (AUC) of 0.9367, further confirming good class separability (Supplementary Fig. 2d). Ninetails can distinguish almost flawlessly between plain adenosine signals and those decorated with non-adenosines (Fig. 1e; Supplementary Figs. 2c, d; Supplementary Data 1). Among the mixed tails, it reports guanosines with high accuracy, close to that of pure adenosines. The model classifies cytidines and uridines with slightly lower accuracy but still above 85%. This can be attributed to the striking similarity of the signatures of these nucleotides (Fig. 1e; Supplementary Fig. 2c, d; Supplementary Data 1).

**Fig. 2:**
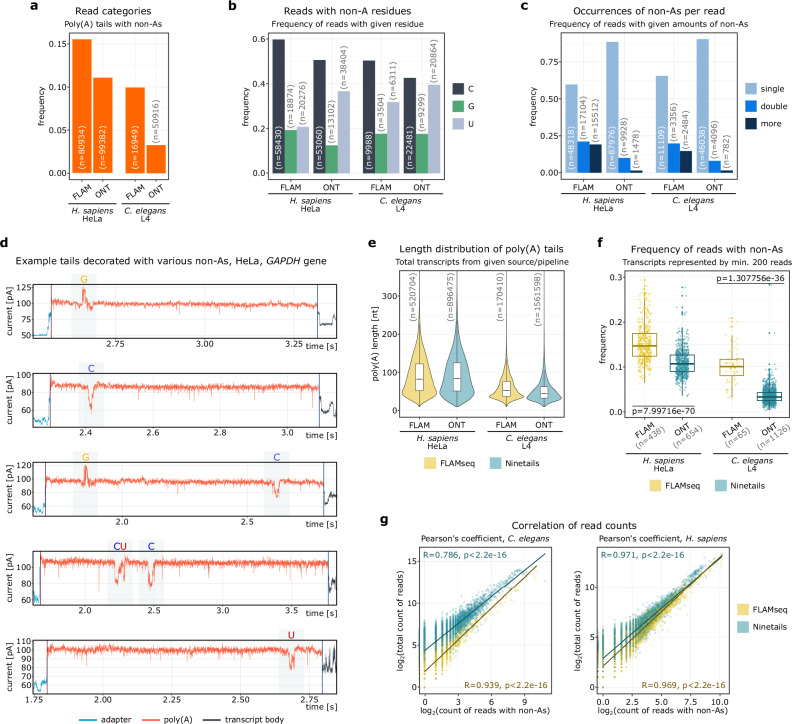
Validation of Ninetails using the orthogonal method (Ninetails vs FLAMAnalysis). **a** Frequency of reads with poly(A) tails decorated with non-adenosines. **b** Frequency of tails with given non-adenosines among all decorated tails. **c** Frequencies of decorated reads with given number of non-adenosines. **d** Example signals corresponding to decorated tails. **e** Poly(A) tail length distribution of either source material. **f** Frequency of decorated tails in transcripts represented by at least 200 reads. *P* values were calculated using Kruskal–Wallis rank sum test, alpha = 0.05. **g** Correlation of the number of decorated reads with the total number of reads in either source material. For panels (**a**–**c**), and (**e**, **f**), FLAM represent result obtained with FLAMAnalysis, ONT – with Ninetails. For each source/pipeline, the Pearson correlation (two-sided) coefficient, *p* value and the linear regression are displayed. In each panel, corresponding read/gene counts (n) are provided. For panels (**e**, **f**) containing boxplot, the horizontal line represents the median, while the lower and upper hinges correspond to the first and third quartiles (25th and 75th percentiles). The whiskers extend to the smallest and largest values within 1.5 times the interquartile range from the hinges.

We then tested the performance of our model on biological data. Since Ninetails is the only tool for nanopore DRS reads, we decided to compare it to FLAMAnalysis, a pipeline designed for state-of-the-art FLAMseq method based on the PacBio sequencing platform^2^. For this purpose, we used PacBio data from *Caenorhabditis elegans* larval stage 4^2^ and HeLa cells^2^, as well as our own *C. elegans’* DRS data from the same developmental stage, and DRS data from HeLa cells obtained from the repository^45^. With this, we juxtaposed key features and trends (Fig. 2).

Ninetails and FLAMAnalysis agree in determining the respective non-adenosine prevalence (Fig. 2a), with cytidine being the most abundant in tails, followed by uridine, and guanosine being the least abundant (Fig. 2b). The two pipelines roughly agree on the frequency of non-adenosines in individual tails. They most often find a single non-adenosine and less frequently two or more (Fig. 2c), with Ninetails identifying fewer tails with non-adenosines relative to the size of the dataset than FLAMAnalysis (Fig. 2a, f). Notably, FLAMAnalysis reports double and multiple insertions more often than Ninetails (Fig. 2c). Since such stretches are not found in the raw nanopore signals, we suspect that they are artifacts of the FLAMseq protocol (Fig. 2d), probably introduced during amplification (Supplementary Fig. 6). Despite this, both approaches provide relatively coherent results (Fig. 2e, g).

To sum up, Ninetails provides a comprehensive analysis of the internal modifications of poly(A) tails, outperforming existing tools that suffer from experimental biases.

### Various enzymes incorporate non-adenosines into poly(A) tails with different prevalence

We next examined the frequency of non-adenosines in poly(A) tails of in vitro synthesized RNAs with different protocols often used to generate prototypes of mRNA therapeutics. We prepared three variants of synthetic RNAs tailed post-transcriptionally by polyadenylate polymerase (PAP) under different conditions: (I) with adenosine triphosphate (ATP) only, (II) with all four ribonucleoside triphosphates (rNTPs) at equimolar concentrations, and (III) with rNTP ratios corresponding to their in vivo stoichiometry^46^. We compared them with two other RNAs, equipped with poly(A) tails encoded in the DNA template and synthesized by either (IV) wild type T7 RNAP or (V) the double mutant G47A + 884 G T7 RNAP developed by Moderna^47^. As previously reported, the latter yields less unwanted immunostimulatory RNA byproducts, which advocates for its prospective use in mRNA therapeutics industry^47^.

**Fig. 3:**
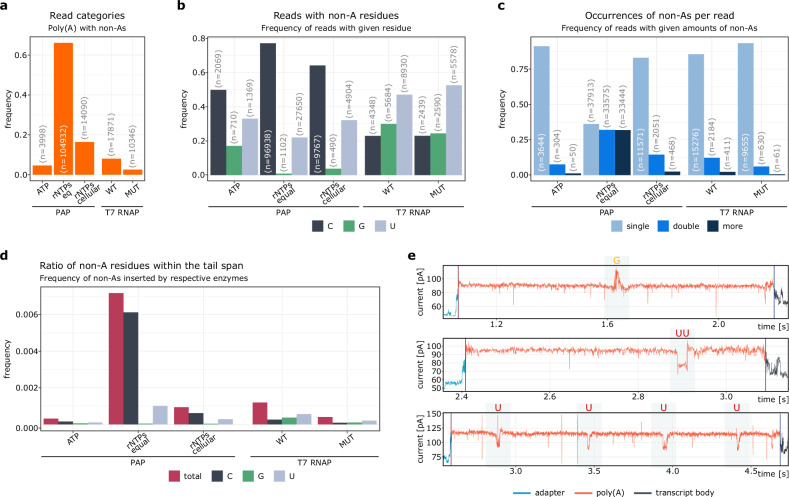
Synthetic RNAs exhibit variable non-adenosine prevalence depending on in vitro synthesis conditions. **a**–**c** Frequency of decorated reads: **a** in tails with given non-adenosines among all decorated tails; **b** in decorated reads with given amount of non-adenosines; **c** in poly(A) tails synthesized using different in vitro approaches. **d** Estimated frequency of non-adenosine nucleotides among the all nucleotide content of synthesized poly(A) tails. **e** Examples of decorated tails. In panels (**a**–**c**), corresponding read counts (n) are provided. Symbols: PAP samples tailed by polyadenylate polymerase, T7 RNAP samples tailed by T7 RNA polymerase (templated), ATP reaction mix with ATP only, rNTPs eq reaction mix with equimolar concentration of rNTPs, rNTPs cellular reaction mix with physiological concentrations of rNTPs, WT wild-type T7 RNAP, MUT double-mutant T7 RNAP.

Poly(A) tails of all analyzed mRNAs were decorated with detectable amounts of non-adenosines (Fig. 3). In PAP-generated tails in conditions corresponding to standard laboratory protocols and physiological conditions, reads with non-adenosines accounted for less than 20% of all reads. However, when equal concentrations of rNTPs were used, nearly 70% of reads equipped with tails synthesized by PAP contained an admixture of non-adenosines (Fig. 3a). In addition, PAP and T7 differ in the prevalence of reads with given non-adenosine inclusions. In samples tailed with PAP, tails with cytidines were the most abundant, followed by those with uridines and guanosines. In molecules synthesized by T7 RNAPs, tails decorated with uridines dominated, followed by tails containing guanosines and cytidines (Fig. 3b). Most tails in mixtures with an excess of ATP (in which the remaining nucleotides were residues from previous steps or had an order of magnitude lower concentration) had a single inclusion. Tails with one, two, or more non-adenosines were evenly distributed when the nucleotide concentrations were equimolar (Fig. 3c). We also found that the frequency of decorated reads did not correlate with the length of poly(A) tails (Supplementary Fig. 7). Tails produced under most experimental conditions contained at most 0.1% non-adenosine and more than 0.7% with PAP at equal amounts of rNTPs (Fig. 3d). All these indicate that the ratio of incorporation of non-adenosines by PAP is significantly affected by the concentration of the respective rNTPs (Fig. 3). This is consistent with previous studies using PacBio (FLAM-seq), and with reports of nucleotide incorporation rates of PAP, confirming the sensitivity and reliability of our method^2,46,48^. We also noticed that the T7 RNAP mutant incorporated only half as many non-adenosines as its’ wild-type counterpart (Fig. 3d). Our findings were confirmed by analysis of the raw nanopore signals (Fig. 3e).

In sum, our data show that synthetic mRNA molecules contain detectable amounts of misinserted non-adenosine residues that can be profiled using our pipeline.

### Poly(A) composition of Moderna mRNA-1273 vaccine changes dynamically in target cells

**Fig. 4:**
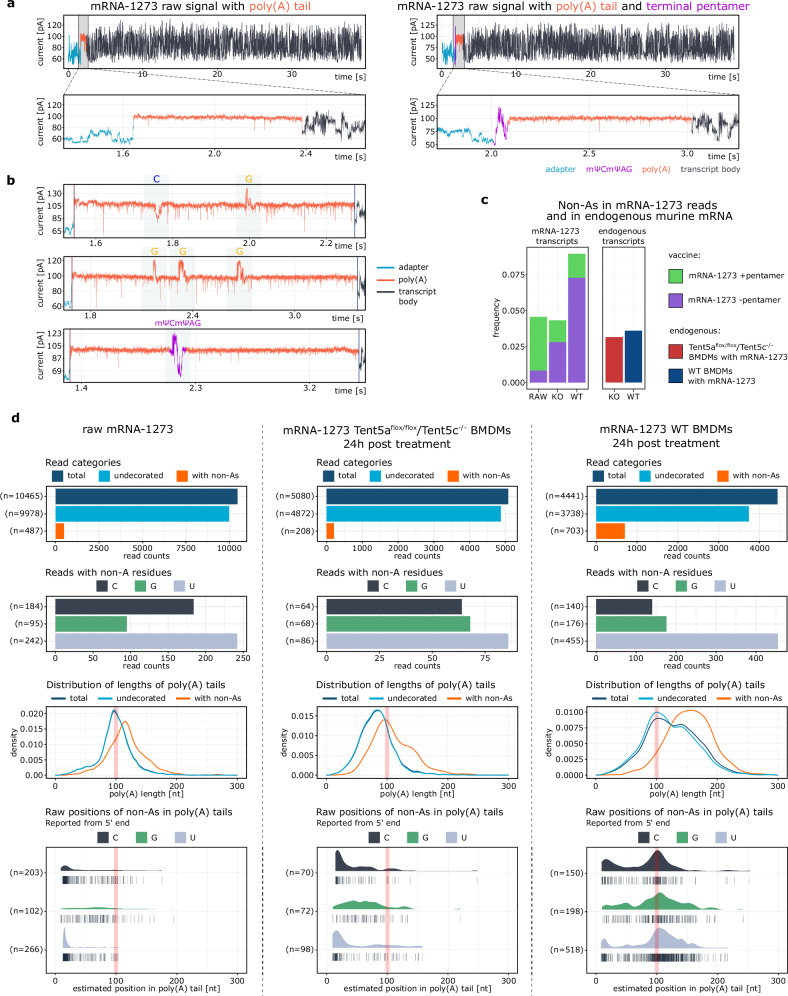
TENT5A activity influences the poly(A) tail composition of Moderna mRNA-1273 Spikevax^TM^ vaccine administered to murine macrophages. **a** Examples of nanopore signals corresponding to mRNA-1273 with (right) and without (left) 3’-terminal mΨCmΨAG. Entire reads shown on top panel, zoomed tail view shown below. **b** Examples of decorated tails in mRNA-1273 reads. At the very bottom, a tail with an internal mΨCmΨAG pentamer (most likely re-adenylated) is shown. **c** Comparison of frequency of decorated tails in mRNA-1273 reads and in endogenous transcripts from BMDMs. Reads ending with the pentamer shown in green, without the pentamer in violet. Data from all time points (4–72 h post-treatment), merged. **d** Detailed comparison of non-adenosine content in crude vaccine and vaccine reads isolated from BMDMs (*Tent5a*^Flox/Flox^*/Tent5c*^*−/−*^ or WT) 24 h after administration. Reads without 3’-terminal mΨCmΨAG pentamer are included. First row: frequency of read categories. Second row: frequency of tails with given non-adenosines. Third row: poly(A) tail length distribution of reads from either category. Fourth row: estimated positions of non-adenosines within poly(A) tails. In third and fourth row the expected position of 3’-terminal mΨCmΨAG pentamer (100 nt) is shown as red rectangle. In figure d first, second and fourth rows, corresponding read counts (n) are provided.

As shown, Ninetails enables comprehensive analysis of the poly(A) tails of endogenous and synthetic mRNAs. Our previous study revealed complex dynamics of the poly(A) tail of the Moderna mRNA-1273 vaccine, which is ∼100 adenosines long, followed by an mΨCmΨAG pentamer^16^ (Fig. 4a). Macrophages are the primary target of this therapeutic mRNA after intramuscular administration. We have shown that mRNA-1273 is re-adenylated in this cell type by TENT5A poly(A) polymerase, which increases the stability and overall efficacy of the vaccine^16^. In an in vitro model of murine macrophages (bone marrow-derived macrophages; BMDMs), 24 h after administration, the poly(A) tails of mRNA-1273 are extended by an average of ∼20 nucleotides, reaching up to 200 nucleotides (Fig. 4b, d). The poly(A) tail length returns to the initial state of ∼100 residues 72 h after delivery. This coincides with the transcriptional induction of TENT5A, followed by a return to its basal level^16^. To gain more insight into the poly(A) dynamics of mRNA-1273 poly(A), we utilized Ninetails. We immediately detected a greater variety of nucleotide content beyond the presence and/or absence of a pentamer at the 3’-end of the tail (Fig. 4b, d; Supplementary Fig. 8a). Scattered non-adenosines (single or multiple per tail) were detected throughout the entire continuity of the tail (Fig. 4b; Supplementary Fig. 8c), both in the crude vaccine and in vaccine reads from treated BMDMs. Uridines were most prevalent, whereas cytidines and guanosines were much less represented (Fig. 4d; Supplementary Fig. 8a, c), which is consistent with the synthetic RNA data presented in the previous section. Interestingly, mRNA-1273 also had a higher percentage of reads containing non-adenosines than endogenous murine transcripts in general (Fig. 4c).

Surprisingly, in some reads we observed the signature of mΨCmΨAG within the poly(A) tail and not only at its terminus (Fig. 4b, c), what indicates that TENT5-mediated re-adenylation may occur independently of the removal of the mΨCmΨAG pentamer^16^. Such a signature was only observed in reads with the elongated poly(A) tail, close to original pentamer location in the tail, 100 nucleotides away from the 3’UTR. However, remnants of the terminal pentamer were found in less than 10% of the re-adenylated tails (Supplementary Fig. 8a), suggesting that either this terminus of the poly(A) tail is not a preferred substrate for TENT5s or that a yet unknown mechanism efficiently removes the pentamer. Interestingly, elongated mRNA-1273 tails were associated with a substantial accumulation of non-adenosines (Fig. 4c, d; Supplementary Fig. 8), which was not observed in cells lacking TENT5A/C polymerases. For poly(A) tails without terminal pentamer, the proportion of reads containing non-adenosines was 1.23% in the crude vaccine, while 24 h after administration it was 5.06% in the mRNA-1273 reads isolated from the double-knockout *Tent5a*^*Flox/Flox*^*/Tent5c*^*−/−*^ cells, and 16.09% in wild-type BMDMs (Fig. 4d). Detailed analysis revealed that this increase was related to the remnants of terminal pentamer, but also to a high incidence of non-adenosines in the elongated part of the tail (Supplementary Fig. 8a, c), suggesting low fidelity of TENT5-mediated re-adenylation.

In summary, the application of Ninetails revealed that the poly(A) tails of mRNA-1273 transcripts are even more heterogeneous than our previous analyses implied^16^. In addition to the terminal mΨCmΨAG, they may also have additional non-adenosines upstream or downstream of the pentamer. Nevertheless, it is currently unknown how the presence of these admixtures affects the stability of the mRNA and, thus, potentially therapeutic outcome.

### Mitochondrial transcripts and TENT5A/C targets are highly decorated with non-adenosines in murine macrophages

**Fig. 5:**
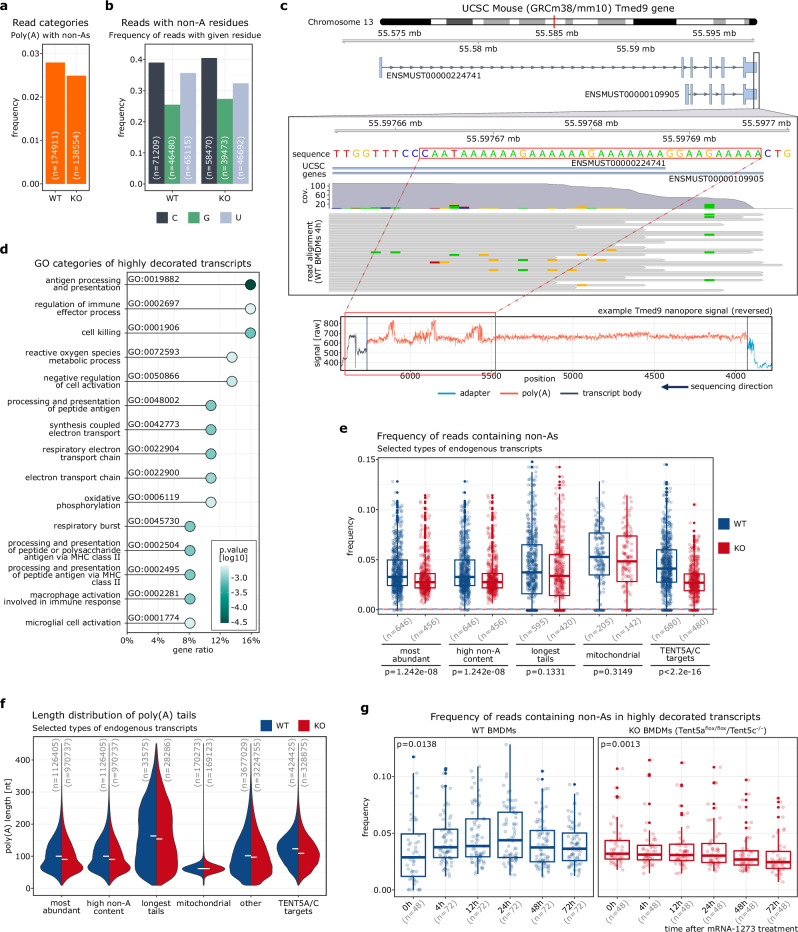
Murine macrophages exhibit a high content of non-adenosines in mitochondrial transcripts and in TENT5A/C substrates. Frequency of decorated reads (**a**) and tails with given non-adenosines (**b**) in murine macrophages transcriptome. **c** Genome-browser plot of reads aligned to 3’UTR of *Tmed9* gene with example of corresponding nanopore signal revealing semi-templated poly(A) tail origin and internal non-adenosines in fixed positions. **d** Top 15 GO terms of highly decorated transcripts (ordered by gene ratio). Only transcripts represented by at least 100 reads per sample are included. Enrichment GO categories for biological processes are shown. The Kolmogorov–Smirnov-like statistic (enrichment score) with Benjamini–Hochberg adjustment, alpha 0.05 is shown as color hue. Frequency of decorated tails (**e**) and poly(A) tail length distribution (**f**) in selected groups of transcripts. On panel (**e**) dashed lines indicate frequencies for the other transcripts (not included in any of selected categories) and p.values between conditions in each group of transcripts were calculated using Mann–Whitney U test, two-sided, alpha = 0.05, with Benjamini–Hochberg adjustment. **g** Frequency of tails containing non-adenosines in highly decorated transcripts of murine macrophages for up to 72 h after mRNA-1273 treatment. *P* values were calculated using Kruskal–Wallis rank sum test, alpha = 0.05. In each panel, corresponding read/gene/observation counts (n) are provided. For panels (**e**, **g**) containing boxplots, the horizontal lines represent the median, while the lower and upper hinges correspond to the first and third quartiles (25th and 75th percentiles). The whiskers extend to the smallest and largest values within 1.5 times the interquartile range from the hinges.

Given the role of TENT5A/C in mRNA-1273 vaccine re-adenylation and non-adenosine incorporation, we investigated the composition of poly(A) tails of murine transcripts in wild-type and double-knockout *Tent5a*^*Flox/Flox*^*/Tent5c*^*−/−*^ BMDMs^16^. The number of reads with tails featuring non-adenosines generally did not exceed 3%. Notably, the overall frequency of decorated reads was slightly higher (0.005%) in wild-type macrophages than in cells lacking TENT5A/C (Fig. 5a). In general, the endogenous poly(A) tails most frequently contained admixtures of cytidines, followed by uridines and guanosines, unlike in the mRNA-1273 vaccine (Fig. 5b; see also Fig. 4d).

In most cases, we detected a single non-adenosine per tail, rarely multiple occurrences of the same or different residues (Supplementary Fig. 9a). Moreover, the non-adenosines were fairly evenly distributed. Nevertheless, for some mRNAs we observed an increased number of non-adenosines in certain regions of the poly(A) tail, especially close to its borders. This was often caused by the misplacement of the poly(A) tail by the nanopolish polya software. Since Ninetails inherited this error, the indicated non-adenosines actually belong to the transcript body or sequencing adapter. We filtered out these artifacts as described in Materials and methods. A characteristic regular pattern of non-adenosine distribution is also found in transcripts with adenosine-rich 3’-untranslated regions (3’UTRs). They have partially templated poly(A) tails, often containing non-adenosines at fixed positions. We have identified 1,035 such transcripts in the murine transcriptome, one of which is *Tmed9* (Fig. 5c). These transcripts allowed us to fine-tune further and validate the efficacy of Ninetails.

To identify functions and processes that might be affected by decorated tails in BMDM, we performed a gene ontology analysis of the transcripts with the highest ratio of tails containing non-adenosines. Figure 5d shows that most decorated transcripts are related to immune response, many of which are known targets of TENT5A/C^16,49^. Specifically, these are effectors of the innate immune response (e.g. *ApoE*, *Cst3*, *Ctsb*, *Ctsd*, *Ctss, Fcgr3, Fth1, Gpnmb, Lrp1, Lyz2*), MHC subunits (*H2-D1, B2m*) and lysosomal proteins (*Lamp1*). We have previously shown that the expression and tail length of many of these immune-related genes increases upon mRNA-1273 uptake and induction of TENT5 poly(A) polymerases^16,49^ (Fig. 5e–g; Supplementary Fig. 9b). Another group contained mitochondrially encoded components of the respiratory chain (e.g. *mt-Atp6, mt-Co1, mt-Co2, mt-Co3*), which are tailed by mtPAP. Finally, the last group of highly decorated RNAs were structural constituents of the ribosome (*Rpl27*, *Rpl38*, *Rpsa*).

Since administration of mRNA-1273 in BMDMs changes gene expression and polyadenylation status^16^, we then questioned whether this also affects the nucleotide composition of poly(A) tails. Therefore, we examined the data from individual time points up to 72 h after the administration of mRNA-1273 vaccine. In contrast to the TENT5-mutant cells, we observed a clear trend in wild-type macrophages. For the most abundant transcripts with a high percentage of composite tails, the proportion of decorated reads gradually increased up to 24 h after vaccination, before declining towards its basal level (Fig. 5g; Supplementary Fig. 9c, d). This trend is similar to that observed for the mRNA-1273 reads. Since it mainly applies to known TENT5 targets, it is likely due to the low fidelity of TENT5-mediated adenylation. This is also supported by the fact that a higher abundance of non-adenosines was observed in the wild-type BMDMs as compared to the mutant cells, as well as an increased admixture frequency after vaccine administration (Supplementary Fig. 9b).

These results suggest that the prevalence of non-adenosines varies between transcripts and that non-adenosine content can be dynamically altered by the action of non-canonical poly(A) polymerases, as it was previously shown for TENT4A/B^12^, and now also for TENT5A.

### Consistent patterns shape nucleotide composition of poly(A) tails in murine cells

**Fig. 6:**
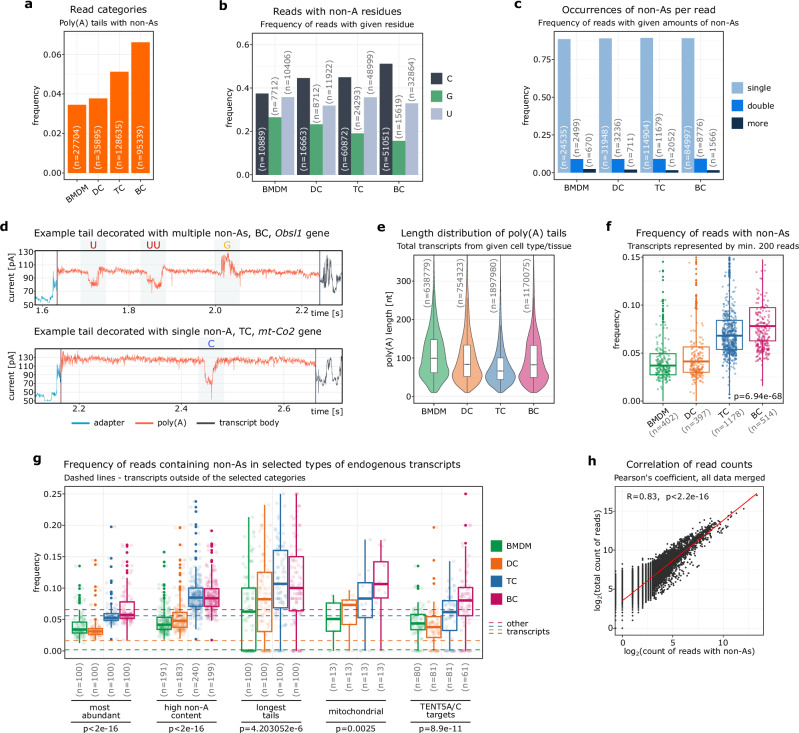
Non-adenosine content varies across murine cell types. Frequency of decorated reads (**a**), tails with given non-adenosines (**b**) and decorated reads with given amount of non-adenosines (**c**) in various murine cells. **d** Examples of decorated tail signals. Poly(A) tail length distribution (**e**) and frequency of decorated tails (**f**) of either cell type. On panel (**f**) only transcripts represented by at least 200 reads are included. *P* values were calculated using Kruskal–Wallis rank sum test, alpha = 0.05. **g** Frequency of decorated tails in selected groups of transcripts. Dashed lines indicate frequencies for the other transcripts (not included in given categories). *P* values between conditions in each group of transcripts were calculated using Kruskal–Wallis rank sum test, alpha = 0.05. **h** Correlation of the number of decorated reads with the total number of reads in all datasets merged. The Pearson correlation (two-sided) coefficient, p.value and the linear regression are displayed. In panels (**a**–**c**, **e**, **f**), corresponding read/gene/observation counts (n) are provided. Sample annotation: BC B cells, BMDM bone marrow-derived macrophages, DC dendritic cells, TC T cells. For panels (**f**, **g**) each dot represents single gene. For panels (**e**–**g**) containing boxplots, the horizontal lines represent the median, while the lower and upper hinges correspond to the first and third quartiles (25th and 75th percentiles). The whiskers extend to the smallest and largest values within 1.5 times the interquartile range from the hinges.

After investigating the complexity of poly(A) tails in BMDMs, we decided to extend our screening to other murine cell types. Therefore, we applied our software to DRS data of B cells (BCs) and T cells (TCs), as well as previously sequenced dendritic cells (DCs)^16^. As shown in Fig. 6a, BMDMs featured the lowest percentage of reads with decorated tails, while BCs contained the highest. We found that cytidine was predominant in all cell types examined, followed by uridine and guanosine (Fig. 6b). Furthermore, decorated poly(A) tails generally contained single non-adenosine, whereas two or more instances were rare (Fig. 6c). Interrogation of the raw signals confirmed these observations. Ninetails correctly identified most non-adenosines, even in relatively short tails (~50 nt). Although it was trained on single nucleotide contexts, it can also recognize adjacent non-adenosines if the signal quality is sufficient (Fig. 6d).

We also investigated whether the frequency of decorated reads correlates with poly(A) tail length, as was observed by Legnini et al. in FLAMseq results^2^. BMDMs had the highest median tail lengths with the lowest median frequency of decorated tails. DCs and BCs had similar median tail lengths, while BCs exceeded DCs in the abundance of non-adenosine tails by almost twofold. TCs, on the other hand, had the shortest tails and slightly less decorated tails than BCs. This suggests a lack of correlation between the global distribution of poly(A) tail lengths and the overall frequency of decorated reads (Fig. 6e, f; see also Fig. 5e, f). We therefore decided to take a closer look at the poly(A) landscape of the examined cells in search of particularly decorated mRNAs (Fig. 6g).

A significant percentage of transcripts in each of the analyzed groups contained an elevated ratio of tails with non-adenosines compared to the ratio of decorated reads in the remaining mRNAs (i.e. not included in these groups) from a given cell type. The most abundant transcripts in BMDMs and DCs have a significantly higher number of decorated reads than the other transcripts. However, this is not the case for BCs and TCs (Fig. 6g). On the other hand, the transcripts with the longest tails in all cell types studied were among the mRNAs with the highest proportion of decorated reads. It is also significant that mitochondrially encoded transcripts with short tails (~50 nt on average) produced by mtPAP and nuclearly encoded TENT5A/C targets had a significantly higher percentage of tails with non-adenosines than all other transcripts (Fig. 6g; see also Fig. 5e, f). This suggests that poly(A) polymerases have varying fidelities.

In all analyzed datasets, we found a strong correlation between the number of decorated reads for a given gene and the total number of reads representing that gene (Fig. 6h). This result is consistent with previous FLAM-seq^2^ results and confirms the reliability of our methodology.

## Discussion

Although DRS has been available for several years, it has not been used for direct profiling of non-adenosines in mRNA due to the lack of methods for processing poly(A) tail regions in nanopore signals. To overcome this limitation, we developed Ninetails, a fast and accurate software that classifies, quantifies, and estimates positions of non-adenosines in poly(A) tails with single-read resolution in DRS data. Our pipeline avoids amplification errors, thereby providing ground-truth level information about the composition of poly(A) tails. A number of methods have already been developed for high-throughput analysis of poly(A) content and length based on PCR-amplified cDNA^2,9–11^. Apart from PCR-based biases, these methods have additional method-specific constraints. Illumina-dependent TAIL-seq combines short-read sequencing with fluorescence quantification. It provides deep coverage and allows estimation of poly(A) tail lengths and investigation of terminal modifications^5,11^. Unfortunately, TAIL-seq does not detect internal non-adenosines, excludes tails longer than ~230 nt from analysis, and is no longer compatible with new Illumina equipment^4,11^. In contrast, FLAM-seq and PAIso-seq use the PacBio third-generation sequencing platform, which handles homopolymers more effectively than Illumina^50,51^. Both methods offer the undeniable benefit of generating long reads with low error rates and thus provide insights into the internal composition of poly(A) tails regardless of their length, unlike TAIL-seq^2,9–11^. At the same time, they overestimate the proportion of non-adenosine stretches.

In this report, we have demonstrated the effectiveness of Ninetails in a variety of synthetic and biological contexts. Our current model can classify and distinguish cytidine, guanosine, and uridine from adenosines (Fig. 1; Supplementary Figs. 1, 2). Ninetails also has the potential to recognize other bases, provided their signatures are sufficiently different from the aforementioned. Ninetails application showed that cytidines were the most enriched in both synthetic constructs and biological samples, followed by uridines and guanosines (Figs. 2b, 3b, 5b, and 6b). Notably, this order reflects the nucleotide specificity of the poly(A) polymerases^48^. We have further shown that the non-adenosine prevalence for Moderna mRNA-1273 was dynamic. Uridine was predominant in the tails of the crude vaccine, followed by cytidine and guanosine (Fig. 4d). While in the tails of the vaccine reads obtained from murine BMDMs, uridine was followed by guanosine and cytidine (Fig. 4d; Supplementary Fig. 8a). This result is mainly related to the fact that TENT5A-mediated re-adenylation may occur in vaccine mRNA that still possesses the mΨCmΨAG terminal pentamer or rather, its remnants within the tail sequence^16^. Further research is needed to determine the extent to which the tail composition affects the stability of therapeutic mRNAs. Understanding this is particularly important when the therapeutic mRNA sector is growing rapidly.

Ninetails analysis confirms that nucleotides other than adenosine are relatively rare in poly(A) tails. A single non-adenosine per tail is most common (Figs. 3c, 4c, and 5c). It appears that the addition of non-adenosines is a stochastic and pervasive process with no clear gene specificity (Figs. 6g and 2g). However, in contrast to previous reports^2,9^, our analyses show that certain groups of transcripts, including mitochondrial mRNAs and TENT5A/C substrates, are particularly rich in non-adenosines compared to the other transcripts (Figs. 5e, 6f; Supplementary Fig. 9). These differences are mainly due to the different fidelities of the cellular poly(A) polymerases. Many TENT5A/C targets are involved in the immune response. Their expression in murine BMDMs increases after vaccination^16^ (Supplementary Fig. 9a). How and to what extent the presence of non-adenosines in the tails of these transcripts affects their fate remains to be investigated, which may be aided by the application of DRS followed by Ninetails data analysis.

## Methods

### In vitro transcription and polyadenylation

Set of transcripts with well-defined poly(A) tail were prepared in vitro by T7 RNAP (WT, mutant: G47A + 884G^47^). Templates for IVT were prepared in consecutive PCR reactions. First, transcript body comprising a fragment of *Renilla* luciferase was amplified from pClneo-NHA plasmid carrying pRL-5BoxB (*Renilla* luciferase containing five BoxB structures) with primers comprising specific to *Renilla* sequence and overhang necessary for PCR2 (RLucX_F1/RLucX_R1) with the following primers:

RLucA_F1: GCCATCAGATTGTGTTTGTTAGTCGCTATGATTCCGAGAAGCACGCCGAGAAC

RLucA_R1: GCTTACGGTTCACTACTCACGACGATGGGACGATGGCCTTGATCTTGTCTTGG

RLucB_F1: GCCATCAGATTGTGTTTGTTAGTCGCTGCTTGTCTGGCCTTTCACTACTCCTACG

RLucB_R1: GCTTACGGTTCACTACTCACGACGATGGTCGGGCTTGCCTCCCTTAACGAGAG

RLucSh_F1: GCCATCAGATTGTGTTTGTTAGTCGCTCTGGAGCCATTCAAGGAGAAG

RLucSh_R1: GCTTACGGTTCACTACTCACGACGATGTTACTGCTCGTTCTTCAGCACGCG

The purified amplicon was a template for PCR2 where T7 promoter sequence on primer add T7_F2 and a variant of poly(A) tail A60_x30_R2/A60_R2 were introduced with the following primers:

addT7_F2: TAATACGACTCACTATAGGGAGAGCCATCAGATTGTGTTTGTTAGTCGCT

A60_c30_R2: TTTTTTTTTTTTTTTTTTTTTTTTTTTTTTGTTTTTTTTTTTTTTTTTTTTTTTTTTTTTGCTTACGGTTCACTACTCACGACGATG

A60_g30_R2: TTTTTTTTTTTTTTTTTTTTTTTTTTTTTTCTTTTTTTTTTTTTTTTTTTTTTTTTTTTTGCTTACGGTTCACTACTCACGACGATG

A60_u30_R2: TTTTTTTTTTTTTTTTTTTTTTTTTTTTTTATTTTTTTTTTTTTTTTTTTTTTTTTTTTTGCTTACGGTTCACTACTCACGACGATG

A60_R2: TTTTTTTTTTTTTTTTTTTTTTTTTTTTTTTTTTTTTTTTTTTTTTTTTTTTTTTTTTTTGCTTACGGTTCACTACTCACGACGATG

In this step, the products with the following sequences were obtained:

RLucB-A60c30: GCTTGTCTGGCCTTTCACTACTCCTACGAGCACCAAGACAAGATCAAGGCCATCGTCCATGCTGAGAGTGTCGTGGACGTGATCGAGTCCTGGGACGAGTGGCCTGACATCGAGGAGGATATCGCCCTGATCAAGAGCGAAGAGGGCGAGAAAATGGTGCTTGAGAATAACTTCTTCGTCGAGACCATGCTCCCAAGCAAGATCATGCGGAAACTGGAGCCTGAGGAGTTCGCTGCCTACCTGGAGCCATTCAAGGAGAAGGGCGAGGTTAGACGGCCTACCCTCTCCTGGCCTCGCGAGATCCCTCTCGTTAAGGGAGGCAAGCCCGACGTAGCAGCACTCATCACTTGGCATTCGTTTTTTTTTTTTTTTTTTTTTTTTTTTTTGTTTTTTTTTTTTTTTTTTTTTTTTTTTTTT

Rluc-sh_A60-g30:

GGAGCCATTCAAGGAGAAGGGCGAGGTTAGACGGCCTACCCTCTCCTGGCCTCGCGAGATCCCTCTCGTTAAGGGAGGCAAGCCCGACGTCGTCCAGATTGTCCGCAACTACAACGCCTACCTTCGGGCCAGCGACGATCTGCCTAAGATGTTCATCGAGTCCGACCCTGGGTTCTTTTCCAACGCTATTGTCGAGGGAGCTAAGAAGTTCCCTAACACCGAGTTCGTGAAGGTGAAGGGCCTCCACTTCAGCCACGAGGACGCTCCAGATGAAATGGGTAAGTACATCAAGAGCTTCGTGGAGCGCGTGCTGAAGAACGAGCAGTAA GTAGCAGCACTCATCACTTGGCATTCGTTTTTTTTTTTTTTTTTTTTTTTTTTTTTCTTTTTTTTTTTTTTTTTTTTTTTTTTTTTT

RlucA-A60_u30:

ATGATTCCGAGAAGCACGCCGAGAACGCCGTGATTTTTCTGCATGGTAACGCTGCCTCCAGCTACCTGTGGAGGCACGTCGTGCCTCACATCGAGCCCGTGGCTAGATGCATCATCCCTGATCTGATCGGAATGGGTAAGTCCGGCAAGAGCGGGAATGGCTCATATCGCCTCCTGGATCACTACAAGTACCTCACCGCTTGGTTCGAGCTGCTGAACCTTCCAAAGAAAATCATCTTTGTGGGCCACGACTGGGGGGCTTGTCTGGCCTTTCACTACTCCTACGAGCACCAAGACAAGATCAAGGCCATCGTCCGTAGCAGCACTCATCACTTGGCATTCGTTTTTTTTTTTTTTTTTTTTTTTTTTTTTATTTTTTTTTTTTTTTTTTTTTTTTTT

RlucA-A60:

ATGATTCCGAGAAGCACGCCGAGAACGCCGTGATTTTTCTGCATGGTAACGCTGCCTCCAGCTACCTGTGGAGGCACGTCGTGCCTCACATCGAGCCCGTGGCTAGATGCATCATCCCTGATCTGATCGGAATGGGTAAGTCCGGCAAGAGCGGGAATGGCTCATATCGCCTCCTGGATCACTACAAGTACCTCACCGCTTGGTTCGAGCTGCTGAACCTTCCAAAGAAAATCATCTTTGTGGGCCACGACTGGGGGGCTTGTCTGGCCTTTCACTACTCCTACGAGCACCAAGACAAGATCAAGGCCATCGTCCGTAGCAGCACTCATCACTTGGCATTCGTTTTTTTTTTTTTTTTTTTTTTTTTTTTTTTTTTTTTTTTTTTTTTTTTTTTTTT

Product of PCR2 was analyzed and purified over agarose gel with GeneJet Gel Extraction Kit (Thermo Fisher Scientific; cat. no: K0691). RNA was prepared at 37 °C for 1.5 h in 50 µl reaction mix containing: 600 pmols template, 1× buffer (200 mM Tris-HCl, 30 mM MgCl_2_, 10 mM spermidine, 50 mM NaCl), 5 µl of rNTPs mix (20 mM each), 5 µl of 100 mM DTT, 0.5 µl of 1% Triton X-100, 80U ribonuclease inhibitor and homemade T7 RNAP. Next, 4U of DNAse TURBO (Ambion; cat. no: AM2238) was added to the reaction mixture and after 15 min the reaction was terminated with EDTA pH 8.0 (final 25 mM). RNA was cleaned on KAPA Pure Beads (Roche; cat. no: KK8001), concentration was assessed with Qubit RNA high sensitivity (Thermo Fisher Scientific; cat. no: Q32852) protocol, visualized by denaturing electrophoresis, and sequenced via nanopore.

### In vitro polyadenylation

RNA for in vitro polyadenylation was transcribed from PCR templates LacZ_F1/R1, prepared similarly as described above, except they were not coding for poly(A) tail. The sequences of primers were as follows:

LacZ_F1:

GCCATCAGATTGTGTTTGTTAGTCGCTATGGTCGTTTTACAACGTCGTGACTG

LacZ_R:

GCTTACGGTTCACTACTCACGACGATGGCCATCAAAAATAATTCGCGTCTGGCC

3’-naked RNAs were transcribed with T7 polymerase as described above, cleaned on KAPA Pure Beads (Roche; cat. no: KK8001) and examined on denaturing gel. 500 ng of RNA was polyadenylated either in presence of ATP only or rNTPs (in equimolar concentration or in ratio CTP:GTP:UTP:ATP 1:3.3:2.7:23^46^) by *Escherichia coli* PAP polymerase (New England Biolabs; cat. no: M0276) in 20 µl reaction for 30 min at 37 °C, according to manufacturer’s protocol. The reaction was stopped with EDTA pH 8.0 (final 10 mM), purified RNA was measured with Qubit RNA high sensitivity (Thermo Fisher Scientific; cat. no: Q32852) protocol, visualized by denaturing electrophoresis, and used to prepare libraries.

### *Caenorhabditis elegans* cultures and RNA extraction

The population of wild-type *C. elegans* strain (N2 Bristol) was synchronized by bleaching of gravid adults and seeding isolated embryos on NGM plates with *E. coli* HB101. Worms were then kept at 20 °C until they reached the L4 stage. Synchronized worms were collected from plates 55 h after the embryos seeding (10 h after entering the L4 stage) and washed three times using 50 mM NaCl. Worm pellet was then suspended in 1 ml of TRIReagent (Sigma-Aldrich; cat. no: T9424), vortexed for 15 min at room temperature and stored in −80 °C before the RNA isolation. Samples were prepared in two independent biological replicates. RNA was isolated following the manufacturer’s instructions and cap-enrichment of mRNA was performed as described previously^30^. Briefly, total RNA was incubated with gluthatione sepharose 4B resin (GE Healthcare; 17-0756-01) bound to purified GST-eIF4E K119A protein for 1 h at RT. Then RNA was washed three times with buffer B (10 mM potassium PB, pH 8.0, 100 mM KCl, 2 mM EDTA, 5% glycerol (Sigma-Aldrich), 0.005% Triton X-100 (Sigma-Aldrich), 6 mM DTT (A&A Biotechnology), and 20 U/mL Ribolock RNase Inhibitor (Thermo Fisher Scientific, EO0381), twice with buffer B supplemented with 0.5 mM GDP (Sigma-Aldrich), and two times with buffer B without GDP. RNA was eluted from the resin by acid phenol:chloroform extraction followed by ethanol precipitation. Nanopore direct RNA sequencing libraries were prepared from 4 µg of cap-enriched mRNA. To increase the efficiency of the sequencing, 150 ng of oligo-(dT)25-enriched mRNA from *Saccharomyces cerevisiae* and 2 ng of standards with predefined poly(A) lengths were added to each library^30^.

### Mice lines

All mice lines were generated by the CRISPR/Cas9-based method at the Genome Engineering Unit (https://geu.iimcb.gov.pl/)^30,31,49^. Briefly, a conditional knockout Tent5a^Flox/Flox^ (C57BL6;CBA-Tent5a^em2IIMCB^/Tar) mouse line was created by insertion of LoxP sites in introns flanking exon 2, which contains triplets encoding the catalytic center of the protein (D144N and D146N). Cas9-generated double-strand breaks in gDNA were targeted using two chimeric sgRNA. *BamHI* or *HindIII* restriction sites were inserted next to LoxP sites to facilitate genotyping. Whereas a knockout Tent5c^*−/−*^ (C57BL6;CBA-Tent5c^em1IIMCB^/Tar) mouse line was created by generating a random indel mutation within the only coding exon of the protein, destroying its catalytic center (Cys88fs26*). Double Tent5a^Flox/Flox^ Tent5c^*−/−*^ mouse line was created by crossing aforementioned Tent5a^Flox/Flox^ conditional knockout with the Tent5c^*−/−*^ knockout line^49^. Desired mutations were confirmed by Sanger sequencing and followed by genotyping with the following primers^31^:

Fw primer for genotyping of Tent5a KO mouse line: CAAGCCTGATTGTGAAGGTG

Rv primer for genotyping of Tent5a KO mouse line: AAGGAAGAGAAGGAAACGCA

Fw primer for genotyping of Tent5c KO mouse line: AGGTCCTGACTGAGGTCGTG

Rv primer for genotyping of Tent5c KO mouse line: TTCCTCAAAATCCCCGTACA

sgRNA1 (Tent5a^Flox/Flox^): TATGGGCGTCACGATCGGGG

sgRNA2 (Tent5a^Flox/Flox^): ACTAATGCGCGTGAGTGGTG

sgRNA (Tent5c^−/−^): CGGCTTGGGTTGCAAAGATC.

Mice were housed in the animal facility of the Faculty of Biology, University of Warsaw, in conventional polypropylene cages containing wood chip bedding supplied with nesting material and paper tubes. A 12/12 h light cycle was maintained in the room, with at least 15 air changes per hour, 55%  ±  10%, relative humidity, and the 22 °C  ±  2 °C temperature. The animals were fed and hydrated *ad libitum* (Labofeed B, Morawski). Regular health monitoring was conducted at the IDEXX laboratory.

All experiments involving animals were approved by the II Local Ethical Committee in Warsaw (decision no: WAW2/71/2021, WAW2/129/2021, WAW2/95/2022, WAW2/127/2022, and WAW2/007/2023) and were performed according to Polish Law (Act no: 653 266/15.01.2015), and in agreement with the corresponding European Union directive.

### Murine B cells cultures and RNA extraction

Isolation and cultivation of primary splenic B cells from 2 wild-type female mice was performed as described in Bilska et al.^30^. Briefly, 12–16 weeks old animals were sacrificed by cervical dislocation. A single-cell suspension of splenocytes was obtained by mechanical tissue disintegration of the spleen through a 70 µm cell strainer. Then, splenocytes were additionally depleted from red blood cells using ACK lysis buffer before separation. Naive B cells were isolated from spleen using immunomagnetic negative selection with EasySep Mouse B Cell Isolation Kit (Stemcell; cat no: 19854) according to the manufacturer’s instructions. Cells were then activated with 20 µg/ml LPS (Sigma-Aldrich; cat. no: L2630). As these cells were used as a control in an unrelated project, they were transduced with pMSCV retroviral construct encoding scrambled shRNA and GFP to determine transduction efficiency. 72 h after transduction, cells were sorted using the CytoFLEX SRT (Beckman) cell sorter to collect only the GFP+ fraction of cells (that were efficiently transduced). 3 mln cells were collected and the cell pellet was resuspended in TRIReagent (Sigma-Aldrich; cat. no: T9424). RNA was isolated according to the manufacturer’s protocol. RNA was then purified using KAPA Pure Beads (Roche; cat. no: KK8001) in 1:1.8 RNA to beads ratio. For direct RNA sequencing, 4.3 µg of total RNA from B cells was mixed with 200 ng of oligo-(dT)25-enriched mRNA from *S. cerevisiae* and standards with predefined poly(A) lengths and used for library preparation. Each sequencing library originates from a single individual.

### Murine T cells cultures and RNA extraction

Wild-type female mouse was sacrificed at age of 12 weeks by cervical dislocation. A single-cell suspension of splenocytes was obtained by mechanical tissue disintegration of the spleen through a 70 µm cell strainer. Then, splenocytes were additionally depleted from red blood cells using ACK lysis buffer before separation. Naive T cells were isolated with EasySep Mouse T Cell Isolation Kit (StemCell; cat. no: 19851) and stimulated with Dynabeads Mouse T-Activator CD3/CD28 Kit (Invitrogen; cat. no: 11452D) according to manufacturer’s instruction. Primary cells were cultured in RPMI 1640 ATCC’s modified (High Glucose, Low Sodium Bicarbonate, HEPES, L-glutamine; Invitrogen; cat. no: A1049101) supplemented with 10% FBS (Invitrogen), 100 nM 2-mercaptoethanol (Sigma-Aldrich), penicillin/streptomycin (Sigma-Aldrich), with 4:1 activator beads to cell ratio. Total RNA was isolated from T cells with TRIReagent (Sigma-Aldrich; cat. no: T9424) according to the manufacturer’s instructions. A 5 µg of EIF4E-enriched mRNA from T cells mixed with 250 ng of oligo-(dT)25-enriched mRNA from *S. cerevisiae* and standards with predefined poly(A) lengths was used for the preparation of library.

### Murine bone marrow-derived macrophages cell cultures and RNA extraction

Primary BMDM cell cultures were established from bone marrow monocytes isolated from 6 *Tent5a*^Flox/Flox^*/Tent5c*^*−/−*^ and 6 wild-type mice. Young adult animals of both sexes (12–25 weeks old) were sacrificed by cervical dislocation, after which femurs and tibias were isolated, and bone marrow was harvested using a centrifugation-based protocol. Material isolated from multiple individuals (siblings of the same sex) was mixed to obtain number of cells sufficient for subsequent analyses. Bone marrow cells were then plated in IMDM medium (Thermo Fisher Scientific; cat. no: 21980065) supplemented with 10% FBS (Gibco), 100 U/ml penicillin/0.1 mg/ml streptomycin solution (Sigma-Aldrich), and 10 ng/ml macrophage colony-stimulating factor (M-CSF, Preprotech; cat. no: 315-02) and cultured at 37 °C in 5% CO_2_. On the day before the vaccine treatment, 0.5–1 × 10^6^ cells were seeded on a 6-well plate in the abovementioned medium. The 1 µl of Moderna mRNA-1273 in original LNPs formulation was diluted in 150 µl of Opti MEM medium (Thermo Fisher Scientific) at RT. After 10 min the mixture was added dropwise to the cells and gently mixed. Cells were harvested at 0, 4, 12, 24, 48 and 72 h time points. Total RNA was isolated from cells with TRIzol (Thermo Fisher Scientific), according to the manufacturer’s instructions. 3.5–5 µg of total mRNA was mixed with 50–200 ng oligo-(dT)25-enriched mRNA from *S. cerevisiae* and standards with predefined poly(A) lengths, followed by library preparation.

### Murine dendritic cells cultures and RNA extraction

Bone marrow was isolated from the femurs of 6 young adult mice (12–16 weeks old) of both sexes. Material isolated from multiple individuals (siblings of the same sex) was mixed to obtain number of cells sufficient for subsequent analyses. The erythrocytes were lysed with ACK buffer. Cells were then resuspended in RMPI medium (10% heat-inactivated FBS, pen/strep, 1% non-essential amino acids, 50 µM β-mercaptoethanol, 1 mM sodium pyruvate, 2 mM L-glutamine), counted and seeded at a density of 1 ml/ml in non-tissue culture treated Petri dishes (Sarstedt) supplemented with GM-CSF 20 ng/ml and IL-4 10 ng/ml ligands (both from Preprotech, 315-03 and 214-14, respectively) and placed at 37 °C in an atmosphere of 5% CO2 in air. On day 4, 5 ml of growth medium containing GM-CSF 20 ng/ml and IL-4 10 ng/ml was added. On day 7, half of the medium was removed, cells were centrifuged, new medium with GM-CSF 20 ng/ml, IL-4 10 ng/ml was added to the collected (centrifuged) cells and returned to the Petri dish. Two days later, loosely adherent cells and cells in suspension were collected. Total RNA was isolated from cells with TRIzol (Thermo Fisher Scientific), according to the manufacturer’s protocol. 3.5–5 µg of total mRNA was mixed with 50–200 ng oligo-(dT)25-enriched mRNA from *S. cerevisiae* and standards with predefined poly(A) lengths, followed by library preparation.

### Reporting on sex

The sex of mice used to derive cell cultures was not considered in the study design or analysis, as these were not germ line cells, whose transcriptome vary significantly by sex. The sex of the worms was also not included as an experimental factor, as the publicly available datasets used for orthogonal validation (FLAMseq)^2^ lack information allowing disaggregation by sex.

### Nanopore direct RNA sequencing

Sequencing libraries were prepared using Direct RNA Sequencing Kit (cat. no: SQK-RNA002) from Oxford Nanopore Technologies (ONT) according to manufacturer’s protocol. The amount and composition of the input material from biological samples are described in the relevant sections. For crude Moderna mRNA-1273 vaccine, up to 0.5 μg of RNA was used for the library preparation. Sequencing was performed using R9.4.1 RevD flow cells on a MinION device, controlled by MinKNOW software (ONT). Raw sequencing data (fast5 files) were deposited at the European Nucleotide Archive (ENA, accession numbers are listed in Supplementary Data 1).

### Basecalling mapping and poly(A) tail delimitation

Raw sequencing reads were basecalled with Guppy, mapped to respective reference using minimap2^52^ (-k 14 -ax map-ont --secondary=no) and processed with samtools^53^ to filter out supplementary alignments and reads mapping to reverse strand (samtools view -b -F 2320). The poly(A) tail lengths and coordinates were determined using nanopolish polya function^18^. The mRNA-1273-originating reads were identified using subsequence Dynamic Time Warping and modified nanopolish polya algorithm as described in Krawczyk et al.^16^. Software versions, references, accession numbers and additional sample characteristics are included in Supplementary Data 1. In subsequent analyses with Ninetails, reads with quality control tag reported by nanopolish as PASS and SUFFCLIP were considered.

### Signal transformation strategy

For each sequenced molecule, two data vectors were extracted from the corresponding basecalled fast5 file: (I) the raw signal (i.e., the digital representation of the sequenced molecule) and (II) moves (i.e., metadata provided by Guppy, describing whether a significant change in ionic current (state) occurs between consecutive k-mers). The region of interest (poly(A) tail) was delimited by the nanopolish polya function. The raw signal was then winsorized to remove sequencing artifacts (cliffs). The move data were then scaled along the corresponding signal based on the number of events and stride. Both vectors (signal and move) were then linearly interpolated to 80% to reduce background noise and increase computational speed. The signal was then scanned using the z-score thresholding algorithm^41^ to identify significantly divergent regions. To reduce the risk of reporting false positives, the following criteria were used to filter out signals potentially containing non-adenosines: (I) signal distortion (anomaly) that was 3.5 standard deviations away from the mean signal values (empirically adjusted), (II) a signal distortion that spanned at least 5 data points (cliff exclusion; empirically adjusted), and (III) the move value corresponding to a distortion ≠ 0 (significant change of state reported by Guppy). Based on the above, the fragments of the preprocessed signal with a fixed length of 100 data points centered on the signal anomaly were selected. If the signal deviation was near the tail terminus, the missing data points were randomly imputed based on the five most frequent values in the entire poly(A) signal. In this way, a set of vectors of equal length without missing values was produced. Each poly(A) signal was then converted into two single-channel (grayscale) GAFs: summation (GASF) and difference (GADF), which were then combined into an array (i.e., a tensor of shape 100 × 100 × 2). The list of these served as an input to the convolutional neural network.

### Training dataset preparation

The classification model was trained using sequencing data corresponding to an in vitro-transcribed molecules equipped with a pure poly(A) tail or containing a single non-adenosine at fixed position. These synthetic RNAs were prepared as described in In vitro transcription and polyadenylation. To create training subsets containing cytidine, guanosine, and uridine, respectively, the fragments of poly(A) signals centered on the signal anomalies were selected as described in Signal transformation strategy. For further information see Supplementary Note 4.

The subset of signals consisting solely of adenosines, on the other hand, was prepared by selection of fragments which do not comply with previously adopted filtering criteria (i.e., devoid of significant signal distortions & corresponding state changes). All data processing steps are implemented in training set creation functions within Ninetails (outside the main analysis workflow). The resulting data were processed to obtain GAF arrays for each class separately, 9440 GAFs were sampled from each class, label-encoded, and shuffled. The resulting dataset containing 37,760 items in total was divided into the 80:10:10 ratio (training:test:validation).

Functions for filtering and transforming training sets are included in Ninetails (in addition to the basic workflow).

### Model architecture

In the VGG-based model used for signal classification in our study, the input image was a tensor of shape 100 × 100 × 2 corresponding to the 100 data points (samples) derived from the original signal before the angular transformation. Our model consists of four blocks with an increasing number of filters (32, 32, 64, 128). The first two convolutional layers use a 5 × 5 kernel with the valid padding to sample wider local information, while subsequent layers use a smaller, 3 × 3 kernel with the same padding. Each block includes an iteration of 2D convolution, batch normalization layers with ReLU activation and He weight initialization, followed by 2D max–pooling and 50% dropout layers. Since the network has to perform a multi-class classification task, an output layer contains 4 nodes and uses the softmax activation function to assign the given image to one of the 4 classes (Supplementary Data 1). The model was fitted using a first-order stochastic gradient descent optimizer with a learning rate of 0.001^54^. The categorical cross-entropy loss function was optimized, and classification accuracy was monitored. The model was built and compiled in R using the keras^55^ package.

### Model training and evaluation

Training included 36 runs in which the following hyperparameters were fine-tuned: batch size (100, 200), dropout (30%, 40%, 50%), activation function (linear, ReLU, GELU), and kernel initializer (He normal, Glorot normal). The network was trained for 20 epochs in each run, with early stopping (patience = 2) and model checkpoint callbacks. The R package tfruns^56^ was used to perform training and select the best model.

To assess the separability of the classes, the ROCs and AUCs were calculated using a one-versus-one approach with the R package pROC^57^, while the confusion matrix and statistics by class were calculated based on the validation data for the selected (best) model using the R package caret^58^.

### Analysis of nucleotide composition of poly(A) tails

The raw classification results were corrected using a Ninetails’ built-in features to mitigate the potential segmentation error inherited from nanopolish software. Ninetails was launched with the option, which allows to filter out non-adenosines located in the tail extremities (qc = TRUE). The data was also filtered based on the normalized distribution of non-adenosines (reclassify_ninetails_data function). If, for a given transcript, the non-adenosine distribution mode was in the first quantile of the tail length, the data for these positions were marked as a potential artifact. Transcripts with 3’UTRs rich in pyrimidines (having semi-templated tails, like the *Tmed9*) were whitelisted. Ninetails’ built-in whitelists include, but are not limited to, human, worm and murine transcripts.

For individual samples/experimental conditions/genes, the frequency of reads containing non-adenosines and the frequency of separate non-adenosine occurrences reported by Ninetails were reported. The sum of reads containing a given non-adenosine was referred to as counts. The sum of separate occurrences of a given non-adenosine was referred to as hits. Frequencies of decorated reads (abundance ratios; AR) were calculated by dividing the sum of reads with non-adenosines (counts) by the total sum of reads representing a given gene/construct. In contrast, non-adenosine frequencies (length ratios; LR) were estimated by dividing the sum of the reported non-adenosine nucleotide positions for a given gene (hits) by the sum of the lengths of all poly(A) tails representing that gene/construct. The above calculations were performed for all non-adenosines combined and for each nucleotide separately (Supplementary Data 2–6). For biological samples, quantification was done at the gene level, unless otherwise stated.

#### Orthogonal validation of Ninetails with FLAManalysis

PacBio sequencing datasets were preprocessed with FLAMAnalysis pipeline (https://github.com/rajewsky-lab/FLAMAnalysis) according to the developers’ instructions^2^. Then, in the R environment, the data were reformatted to enable comparison with Ninetails outputs. Specifically, a set of custom functions was used to reverse complement the poly(A) tail sequences, generate a separate table of non-adenosine residues, count the frequencies of decorated reads (counts) and individual non-adenosines (hits). The resulting data were then merged with data produced by Ninetails. Based on the results of mapping to reference sequences, a subset of transcripts obtained with both platforms (ONT and PacBio) was selected, separately for HeLa cells and for *C. elegans*. This set was subjected to downstream analyses.

#### Non-adenosine profiling in Moderna mRNA-1273

Sequencing data from Moderna mRNA-1273 read samples (raw and from treated macrophages) were processed as described in detail by Krawczyk et al.^16^. Reads mapping to the Moderna mRNA-1237 sequence and unmapped reads with sufficient agreement with the reference nanopore signal were considered vaccine-derived reads. Tails longer than 100 nt and lacking terminal pentamer directly in the vicinity of the sequencing adapter were considered most likely re-adenylated.

#### Non-adenosine profiling in murine macrophages

The 100 most abundant transcripts were selected from transcripts represented by at least 300 reads in each sample (38 genes). The transcripts with the highest non-adenosine content were considered the top 100 transcripts with the highest combined non-adenosine length ratio among the transcripts represented with at least 300 reads in each sample (38 genes). The transcripts with the longest tails were selected as those with the longest average poly(A) tail lengths among those represented by at least 10 reads in each sample (35 genes). TENT5A/C substrates were indicated by statistically significant differences in adenylation between wild-type and *Tent5a*^Flox/Flox^*/Tent5c*^*−/−*^ double knockout. P.values were estimated using the Mann–Whitney U test (two-tailed, alpha = 0.05) for samples untreated with the mRNA-1273, taking into consideration transcripts represented by at least 10 reads (40 genes). P.values were then adjusted for multiple comparisons using the Benjamini–Hochberg method. A NanoTail package^59^ was used to perform statistical inference. Protein-coding transcripts encoded in the mitochondrial genome were also scrutinized (13 genes).

Transcripts with significantly different non-adenosine content between wild-type and *Tent5a*^Flox/Flox^*/Tent5c*^*−/−*^ mutant were indicated by Fischer’s exact test (alpha = 0.05). Only those represented by at least 10 reads in each group were taken into consideration. The false discovery rate was controlled using the Benjamini–Hochberg method. Calculations were done with the Ninetails software. The test was performed for samples untreated with the mRNA-1273. By combining all statistically significant transcripts, a final set of genes was determined. For these genes, changes in non-adenosine content over time after mRNA-1273 delivery were analyzed. Gene Ontology enrichment analysis was done with g:Profiler^60^.

#### Non-adenosine profiling in various murine cells

The 100 most abundant transcripts were selected from transcripts represented by at least 300 reads in given cell type (group). The transcripts with the highest non-adenosine content were considered the top 100 transcripts with the highest combined non-adenosine length ratio among the transcripts represented with at least 300 reads in given cell type (group). The transcripts with the longest tails were considered the top 100 transcripts with the longest average poly(A) tail lengths among those represented by at least 10 reads in given cell type (group). TENT5A/C substrates for BMDMs were determined based on the statistical inference described in previous paragraph, whereas for the remaining cell types were selected according to the literature data^16,30,49^.

### Statistics and reproducibility

The sample size was not determined by a statistical method. Statistical analysis was performed using data from two or more biologically independent replicates. Statistical analysis of quantitative data was performed using the R environment. The statistical tests used in each case are described in the figure legends. Data were tested for normality using the Shapiro–Wilk test. Most experiments were repeated at least twice, yielding comparable results.

### Data visualization

The results of the poly(A) nucleotide composition analyses were visualized with Ninetails’ built-in plotting functions, based on the ggplot2^61^ and dplyr^62^ packages. Heatmaps were drawn with the ComplexHeatmap^63^ package. Genome browser-like view of *Tmed9* gene was plotted with the Gviz^64^ package. The remaining plots were prepared using ggplot2.

### Reporting summary

Further information on research design is available in the Nature Portfolio Reporting Summary linked to this article.

## Supplementary information

Supplementary Information

Description of Additional Supplementary Files

Dataset 1

Dataset 2

Dataset 3

Dataset 4

Dataset 5

Dataset 6

Reporting Summary

Transparent Peer Review file

## Data Availability

The raw fast5 DRS data generated in this study have been deposited in the European Nucleotide Archive (ENA) database under accession code PRJEB67899 and PRJEB53190. The third-party PacBio sequencing data referenced in this study were obtained from Gene Expression Omnibus (GEO), where had been deposited under accession number GSE126465^2^ [https://www.ncbi.nlm.nih.gov/geo/query/acc.cgi?acc=GSE126465]. The third-party nanopore DRS data referenced in this study were obtained from Sequence Read Archive (SRA), where had been deposited under accession number PRJNA777450^45^ [https://www.ncbi.nlm.nih.gov/bioproject/PRJNA777450]. The metadata of all sequencing runs used in this study are provided in the Supplementary Data 1. The processed data generated in this study (source data) are available in the Supplementary Data 2–6. Ninetails outputs and FLAManalysis outputs used for orthogonal validation of our software (grouped by source/organism/pipeline), Supplementary Data and Supplementary Information are publicly available on Zenodo^65^ [10.5281/zenodo.13310034]. Any additional information regarding data reported in this paper is available from the corresponding author upon request.

## Code availability

The source code of the Ninetails software used for data analysis is publicly available: deposited in Github [https://github.com/LRB-IIMCB/ninetails] repository and in Zenodo^66^ [10.5281/zenodo.13309819]. Detailed user manual is also available on Github [https://github.com/LRB-IIMCB/ninetails/wiki]. Additional code and docker build are also provided on Github [https://github.com/LRB-IIMCB/ninetails_processing] and Zenodo^65^ [https://zenodo.org/doi/10.5281/zenodo.13310034].
