# Supplementary material for: Direct profiling of non-adenosines in poly(A) tails of endogenous and therapeutic mRNAs with Ninetails
Source: Nat Commun. 2025 Mar 18;16:2664. doi: 10.1038/s41467-025-57787-6 (PMC11920217; doi:10.1038/s41467-025-57787-6)
Supplement: Supplementary file 1 — Supplementary Information [file 41467_2025_57787_MOESM1_ESM.pdf]

Supplementary Information

for the manuscript entitled
Direct profiling of non-adenosines in poly(A) tails of endogenous and therapeutic mRNAs with Ninetails

Natalia Gumińska, Katarzyna Matylla-Kulińska, Paweł S. Krawczyk, Michał Maj, Wiktoria Orzeł, Zuzanna Mackiewicz, Aleksandra Brouze, Seweryn Mroczek, Andrzej Dziembowski

Table of contents

Supplementary Figure 1 2

Supplementary Figure 2 3

Supplementary Note 1 Choice of the poly(A) delimitation strategy 5

Supplementary Figure 3 6

Supplementary Note 2 TailfindR compatibility 8

Supplementary Note 3 Signal denoising strategy 9

Supplementary Figure 4 10

Supplementary Note 4 Z-score thresholding algorithm 11

Supplementary Figure 5 12

Supplementary Note 5 Analysis of the other nucleotide contexts 14

Supplementary Figure 6 15

Supplementary Figure 7 17

Supplementary Figure 8 18

Supplementary Figure 9 20

Supplementary References 22

Supplementary Figure 1

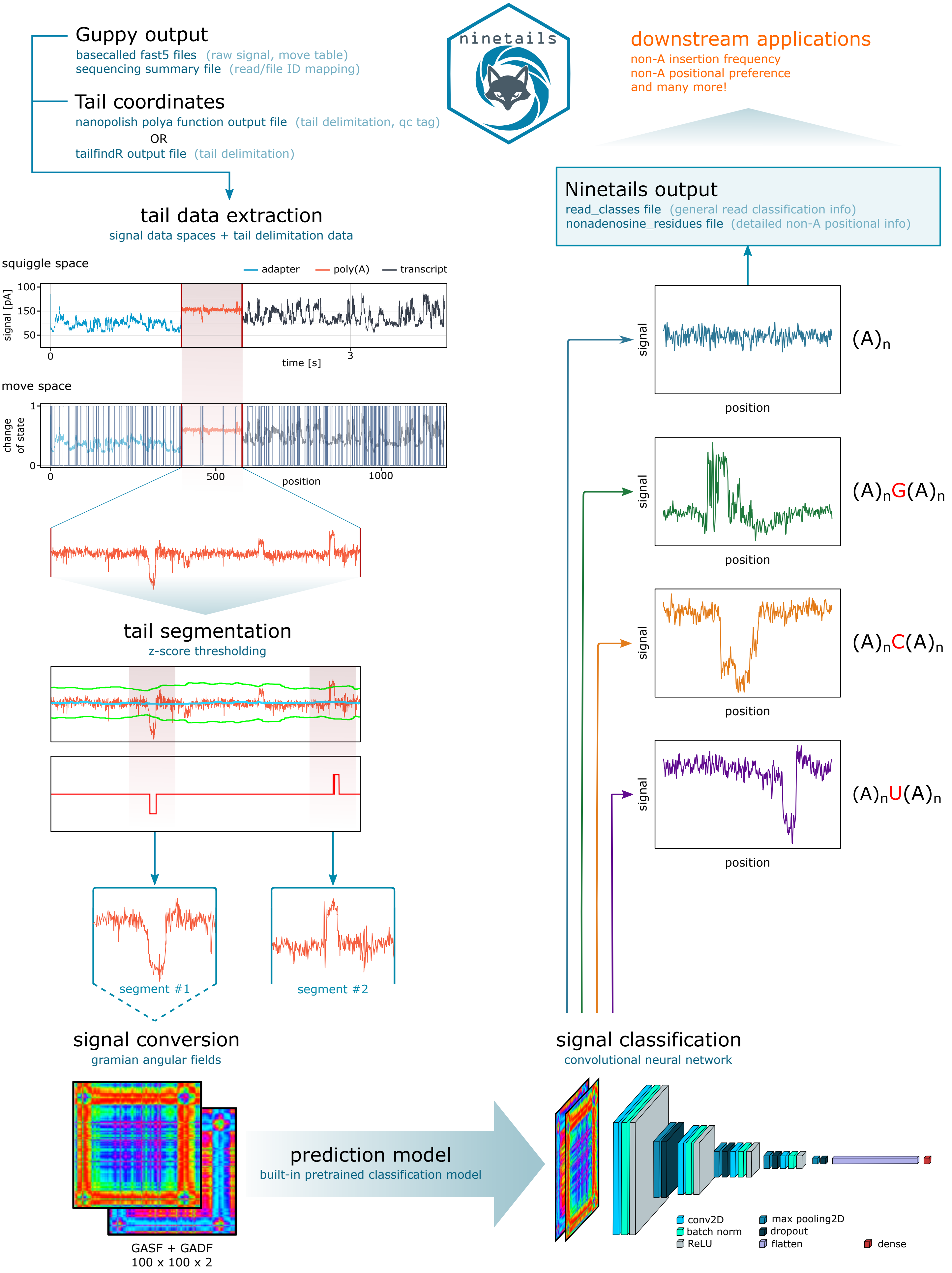

Supplementary Figure 1. The Ninetails workflow scheme.

Schematic representation of the core module of our software - poly(A) tail analyzer and classifier.

Supplementary Figure 2

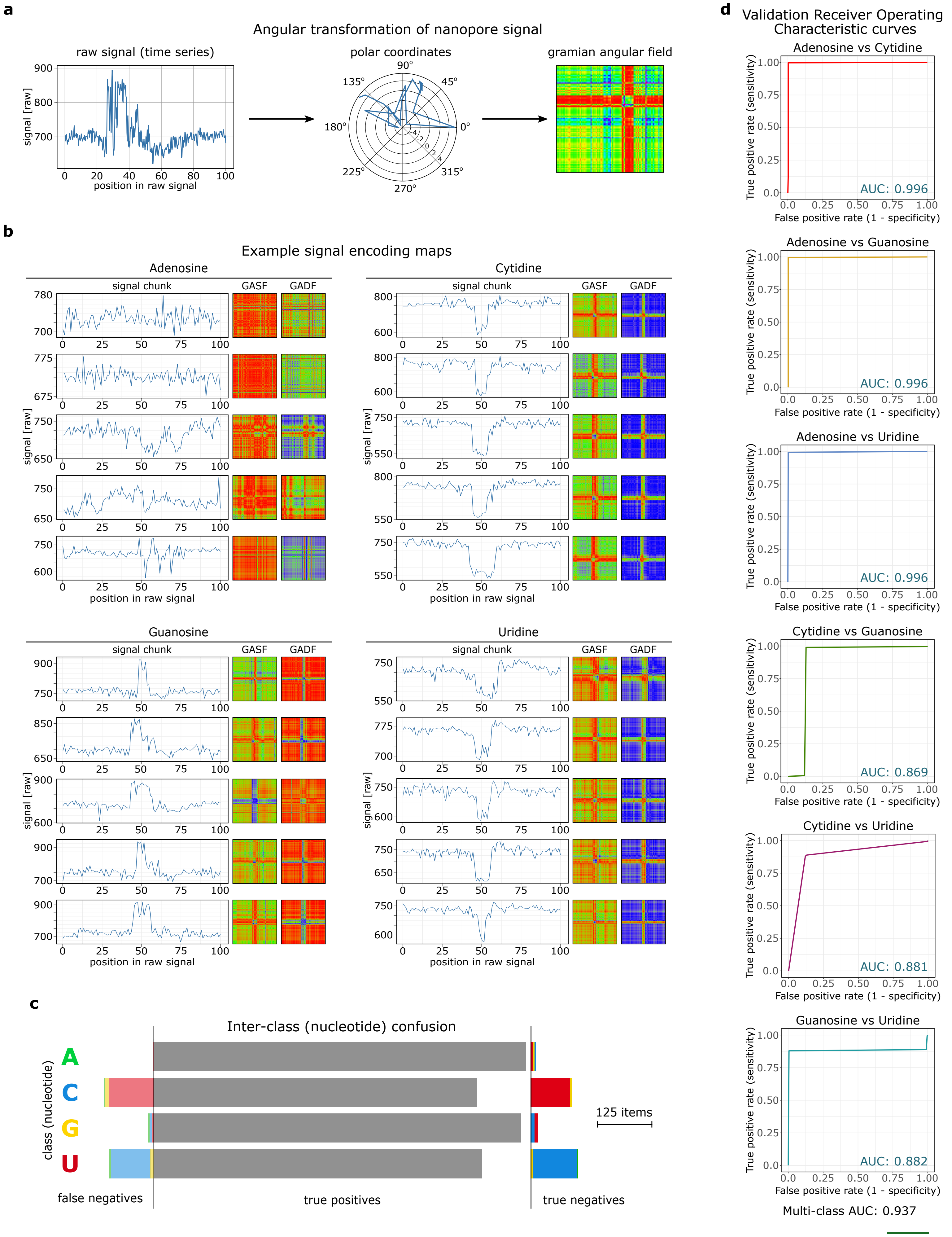

Supplementary Figure 2. Signal transformation and extended validation of model performance.

a, Schematic depiction of angular transformation of nanopore signal. **b**, Examples of nanopore signals with corresponding encoding maps: GAFs and GADFs, respectively. **c**, Visualization of inter-class confusion matrix with emphasis on nucleotide recognition. Confused nucleotides are color-coded (adenosine – green, cytosine – blue, guanosine – yellow, uridine - red), while true positives, regardless of their class, are marked in gray. **d**, Validation receiver operating characteristic (ROC) curves calculated using a 'one versus one' approach. Areas under the curves (AUC) for each compared pair classes and combined multi-class are shown

Supplementary Note 1 Choice of the poly(A) delimitation strategy

Using Nanopore DRS data, poly(A) tails can be delimited, and their length distribution examined. The most widely recognized tools for this purpose are nanopolish and tailfindR. Although both can measure poly(A) tails, neither one provides information regarding their nucleotide content (i.e. the presence of non-adenosines).

Nanopolish is a C++ suite of programs for interacting with Nanopore sequencing data. Its polya function estimates the number of bases in poly(A) tails using a predictive model that combines a Hidden Markov Model (HMM) with an estimator of the translocation rate through the pore. The HMM segments the raw signal into distinct regions (e.g. adapter, poly(A) tail, and transcript body) appearing sequentially as the molecule passes through the pore. Nanopolish requires prior basecalling and downstream data processing (e.g. sequence alignment)[9].

In contrast, tailfindR is an R package that identifies poly(A) tail by thresholding to normalized squiggle, delimiting poly(A) boundaries based on the slope of the raw signal, and normalizing the output by the read-specific nucleotide translocation rate. It relies solely on basecalled reads and requires no further data processing[10].

Both software were benchmarked for accuracy. According to published reports, their outputs are fairly consistent[10,11]. However, we found that tailfindR misidentifies the poly(A) tail region more often than nanopolish, and misplaces its boundaries more frequently. There are instances where tailfindR fails to provide an accurate delimitation of decorated tails, especially those containing non-adenosines near the tail/adapter boundary: it only identifies the portion of the tail preceding or following non-adenosine (Supplementary Figure 3). Additionally, unlike tailfindR, nanopolish performs signal quality assessment, allowing to omit unreliable signals from classification. Finally, computational efficiency of tailfindR is significantly lower compared to nanopolish.

Taking into account all abovementioned concerns, we regard nanopolish as more reliable tool and have therefore selected it as the default source of poly(A) coordinates for Ninetails.

Supplementary Figure 3

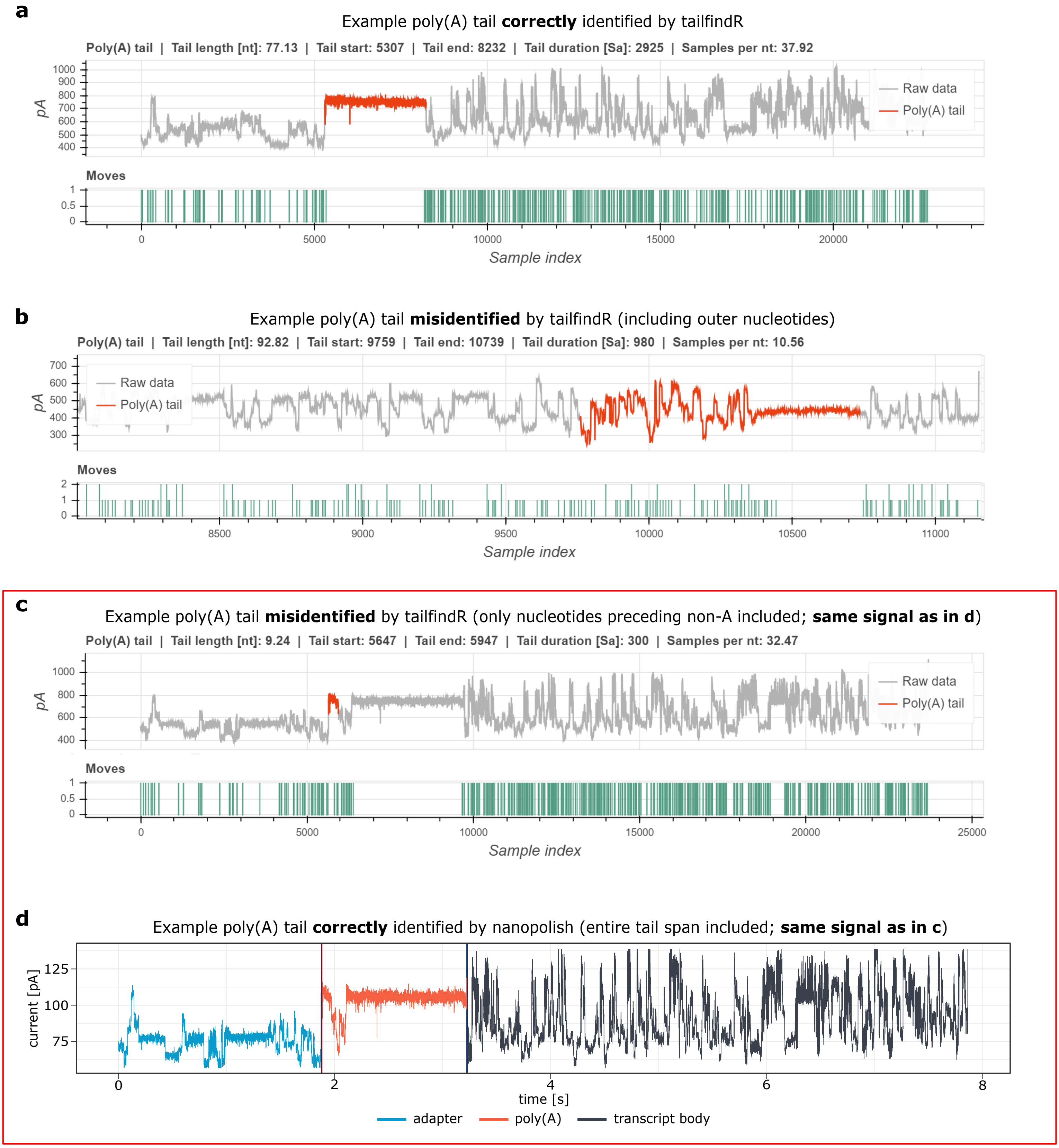

Supplementary Figure 3. Comparison of tail identification by tailfindR and nanopolish.

a, Example signal with blank poly(A) tail correctly identified by tailfindR. **b**, Example signal with blank poly(A) tail misidentified by tailfindR (left coordinate exceeds poly(A) region). **c**, Example signal with decorated poly(A) tail misidentified by tailfindR (only segment between sequencing adapter and the non-adenosine is recognized as poly(A) tail). In a, b, and c, the plots produced with tailfindR are shown. The poly(A) predictions are marked in orange. Corresponding moves are plotted below each signal. **d**, Example signal, the same as in c, with poly(A) tail coordinates delimited by nanopolish. Plot produced with Ninetails is shown. The poly(A) is marked in orange. Vertical lines (maroon and navy blue) denote poly(A) tail boundaries. The red frame contains plots of the same signal (c, d).

Supplementary Note 2 TailfindR compatibility

By default, Ninetails works with the output of the nanopolish polya function: it utilizes poly(A) tail coordinates (to extract the signal area of interest) and quality tags (to consider only those signals whose noise-to-information ratio allows for reliable classification). TailfindR produces similar results, as it also provides tail coordinates, but without signal quality information. Without this knowledge, Ninetails cannot accurately filter sequencing data during the initial analysis step. This in turn can compromise the classification result (i.e. processing signals with excessive noise-to-information ratio may lead to false positives).

Nevertheless, to accommodate needs of the users, we have introduced the possibility of utilizing files generated by tailfindR. The latest version of Ninetails available on Github (v.1.0.2) contains a dedicated function, called *convert_tailfindr_output*. This function allows preprocessing of tailfindR results before passing them to the *check_tails* pipeline wrapper. Specifically, it renames the key columns according to the adopted convention and creates additional required columns. Instructions for its use are provided in the built-in manual and in the Wiki (https://github.com/LRB-IIMCB/ninetails/wiki/8.-TailfindR--compatibility). Users wishing to work with older versions of Ninetails should manually modify tailfindR result files or copy this function from the repository, then load it into the R environment.

However, due to the concerns raised herein (see Supplementary Note 1, Supplementary Figure 3), we strongly recommend the use of nanopolish outputs over tailfindR to mitigate potential bias.

Supplementary Note 3 Signal denoising strategy

We aimed to develop a stable tool with minimal dependencies, efficient data processing, and thorough documentation, that could be widely adopted beyond our research group. Despite the substantial differences in computational capacity between desktops and servers, Ninetails was designed to run on both. Since R stores and manipulates data in the physical memory of the computer (i.e. the RAM), it was necessary to achieve a compromise between the information content of processed objects and their size.

The raw nanopore signal is a noisy time series. It can contain abrupt individual outliers (so-called "cliffs"), especially pronounced in homopolymer regions (e.g. poly(A) tail). Therefore, an important step of feature engineering was the initial preparation of the signal, which is then transformed into an input for the neural network.

We investigated numerous strategies for denoising and rescaling the signal. Our assessment covered a wide range of datasets, including synthetic and biological samples. An example comparison of various methods applied to nanopore signal is provided in Supplementary Figure 2. Ultimately, we adopted winsorization and interpolation. The former removes 5% of the data points which truncates "cliffs". The latter downsamples the signal reducing the background and simultaneously decreasing the load on computer's memory. According to our observations, these manipulations leave the height, width and position of potential non-adenosine signatures (relevant informations) within the signal virtually unaffected. Thus, they do not increase the risk of misclassification.

Very similar effect can be achieved using an R implementation of the Savitsky-Golay filter from the pracma package. However, it introduces terminal artifacts in the signal, which raisies our concerns. Removing them would involve computational resources, and could interfere with subsequent estimation of non-adenosine positions in certain fraction of signals. Therefore, we opted for our own implementation.

Comparable approaches were used in other reports focused on processing nanopore signals (see Supplementary References).

Supplementary Figure 4

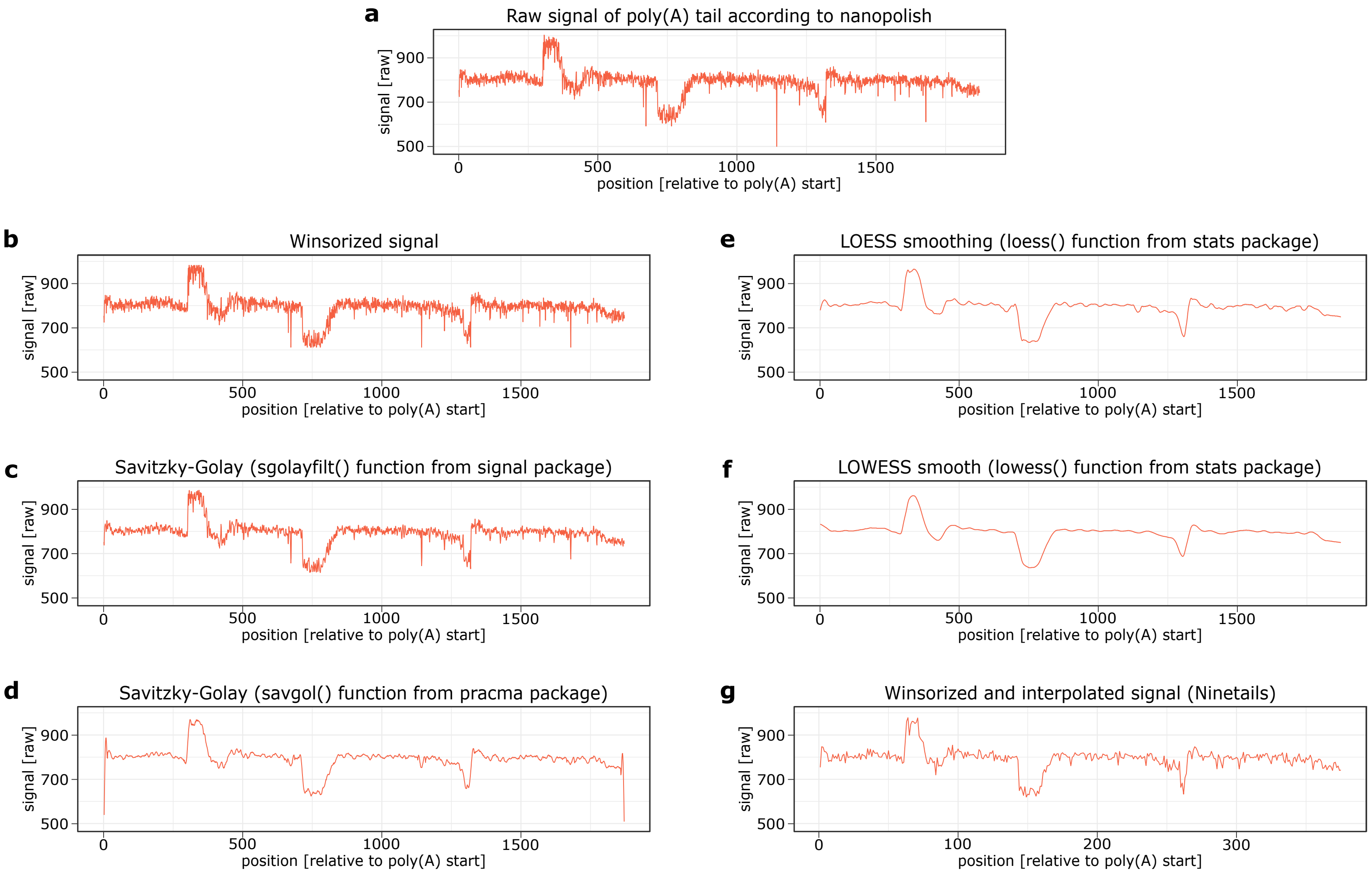

Supplementary Figure 4. Poly(A) tail signal: raw and with various filters applied.

This example signal represents a synthetic molecule with a 60 nt long poly(A) tail containing a single C, G and U every 15 As. The visualization features the same poly(A) tail signal as Supplementary Figure 5. **a**, Raw signal with segmentation provided by nanopolish. **b**, Winsorized signal. **c**, Signal smoothed with Savitzky-Golay filter as implemented in the signal package (default). **d**, Signal smoothed with Savitzky-Golay filter as implemented in the pracma package. **e**, Signal with LOESS (locally estimated scatterplot smoothing) regression applied. **f**, Signal with LOWESS (locally weighted scatterplot smoothing) regression applied. **g**, Winsorized and interpolated signal as adopted in Ninetails with base R functions. All of the applied smoothing filters (b-g) were run using equivalent parameters to ensure comparability.

Supplementary Note 4 Z-score thresholding algorithm

Ninetails initially examines the poly(A) signal for regions exhibiting distortions that are likely to correspond to non-adenosines. During this screening, our rendition of peak-calling algorithm originally developed by van Brakel is used[1]. This algorithm has been implemented in a variety of programming languages and has been used in numerous scientific reports concerning noisy time series[2–8]. In the version adopted in Ninetails, the algorithm reports a peak or valley when a new datapoint is a 3.5 standard deviations away from the moving average (original version does not report valleys). As a result of constructing a separate moving average and a deviation, the algorithm is very robust. At first, it calibrates on values sampled from the entire signal, so initial data points are not omitted from the analysis due to the necessity of filter adjustment. Then it uses an adaptive sampling window of 100 consecutive data points. The algorithm produces a vector of "pseudomoves" with values falling in the range [-1:1], where -1 stands for a valley, 0 for no significant change and 1 for the peak. Subsequently, the Nintails extracts areas of interest from the winsorized and interpolated signal, and after further transformation passes them to the neural network for classification.

Initially, we intended to rely on the values from the move vector (the shifts in raw data representing potential nucleotide translocations provided by default basecaller, Guppy). However, we noticed that the moves do not precisely match the anomalies (Supplementary Figure 3). The changes of states reported by Guppy are shifted from the center of the anomaly by a stochastic value, which impedes precise extraction of the entire anomaly and subsequent estimation of the nucleotide position of detected non-adenosine. Furthermore, Guppy often records more than one instance of a state change within a single anomaly. Finally, it sometimes reports changes of states where the anomaly is absent (false positives). Thus, we used the thresholding algorithm as move vector correction strategy to avoid potential data biasing.

Supplementary Figure 5

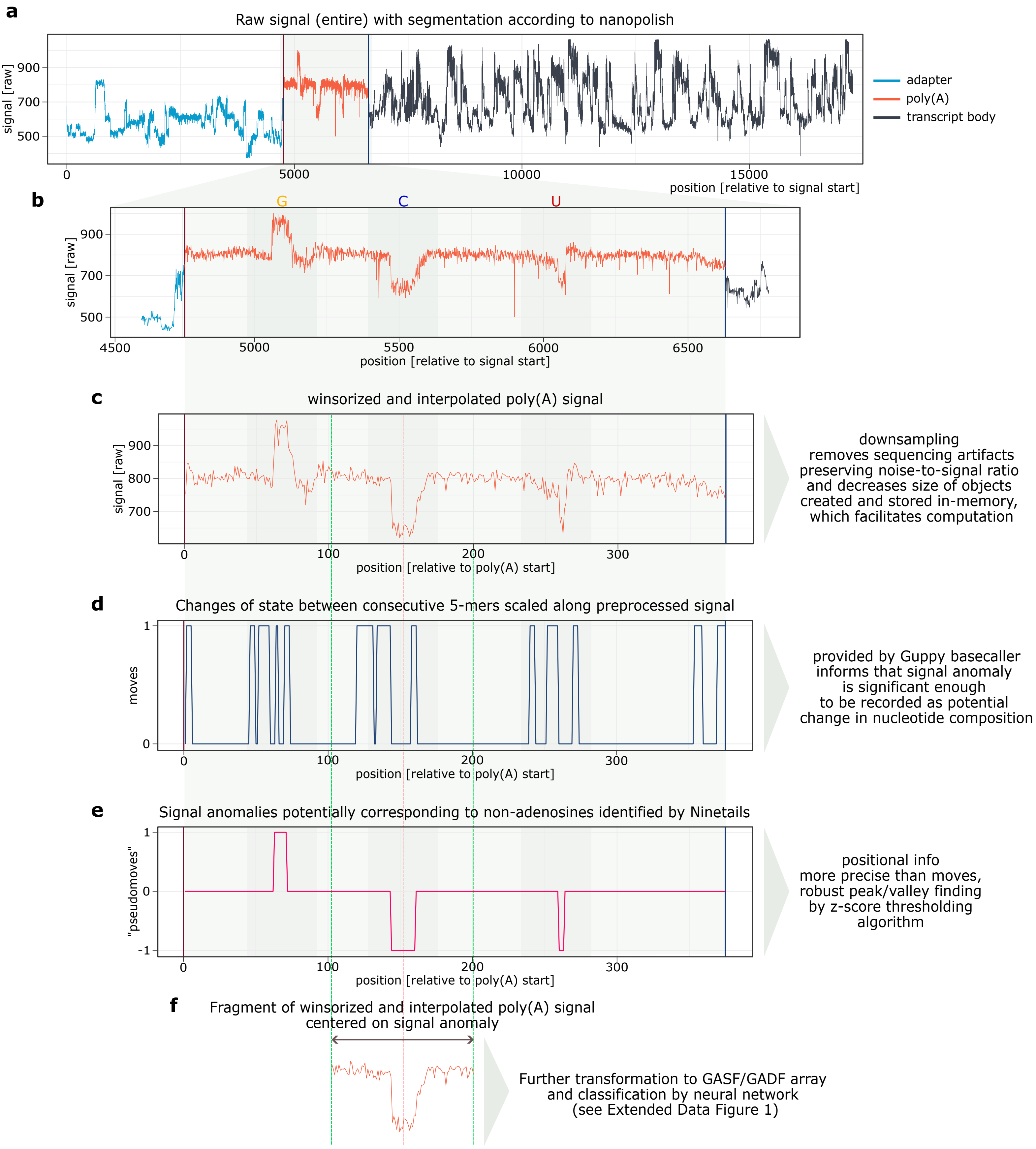

Supplementary Figure 5. Signal downsampling and selection of poly(A) fragments potentially decorated with non-adenosines.

This example signal represents a synthetic molecule with a 60 nt long poly(A) tail containing a single C, G and U every 15 As. The visualization features the same poly(A) tail signal as Supplementary Figure 4. **a**, Raw signal with segmentation provided by nanopolish. **b**, Close-up view of the poly(A) tail region. For easier understanding, the signatures of individual non-adenosines are labeled and marked with gray background. **c**, Poly(A) tail signal after initial preprocessing by Ninetails: winsorized and interpolated. **d**, Graphic representation of corresponding move vector (extracted from fast5 file basecalled by Guppy) scaled along the preprocessed signal. **e**, Graphic representation of the significant peaks/valleys detection by the thresholding algorithm ("pseudomoves"). **e**, An example of an extracted signal chunk with an anomaly in the center. Only one of the three is shown to keep the figure clear

Supplementary Note 5 Analysis of the other nucleotide contexts

Given the technical limitations of nanopore sequencing, noisy character of obtained signal and our desire to create a lightweight yet reliable classifier, we trained our default model on single non-adenosine context. Single non-adenosine nucleotides separated by an adenosine stretch is the most common variant in real-life data. However, we realize that other arrangements may also occur in nature. Our current default model sometimes recognizes adjacent non-adenosine as separate events. Yet it should be treated with caution, since it is not the rule. The classification outcome depends largely on the quality of the signal.

Our strategy was a trade-off between reporting accuracy and minimizing false positives. We offer a pretrained built-in model which recognizes 4 basic non-adenosine nucleotides surrounded by adenosine context. However, Ninetails offers flexibility beyond its default model. It contains set of functions and detailed instructions enabling users to construct custom models incorporating diverse nucleotide contexts. All required informations are available in built-in help, repository on Github (https://github.com/LRB-IIMCB/ninetails_processing/) and upon request.

Supplementary Figure 6

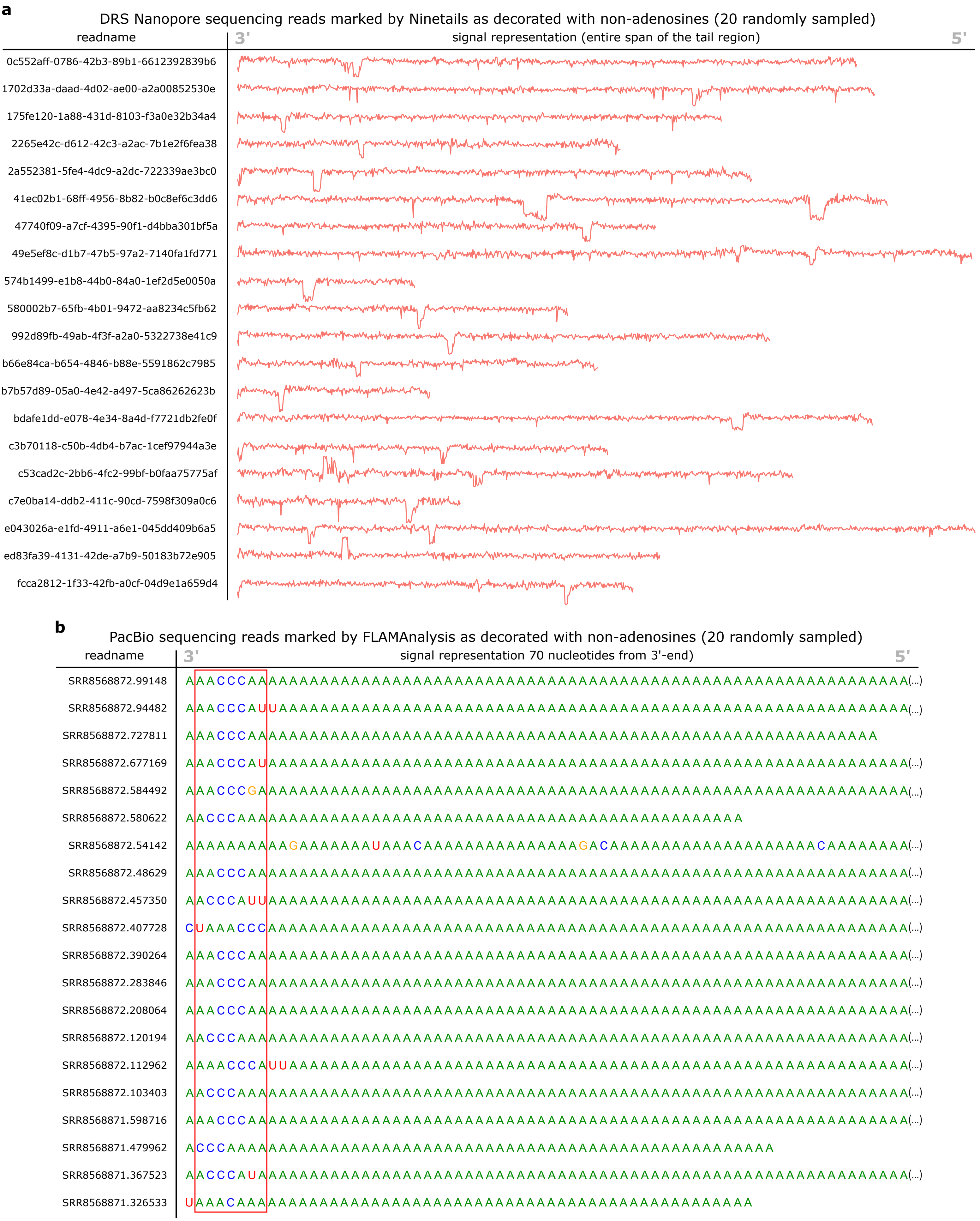

panels a and b are not scaled to the single nucleotide resulution due to readability reasons

Supplementary Figure 6. Comparison of example decorated DRS signals with FLAMAnalysis output revealing the presence of potential PacBio artifacts.

a, Representation of poly(A) tail signals marked by Ninetails as decorated with non-adenosines. 20 decorated reads corresponding to the HeLa *Rpl23* gene were randomly sampled from the Ninetails output. The signals were aligned to the 3'-end. Values and scales are omitted due to readability reasons. Signal length proportions are preserved. **b**, Representation of 20 decorated poly(A) tails according to FLAMseq. 20 decorated reads corresponding to the HeLa *Rpl23* gene were randomly sampled from the FLAMAnalysis output. The sequences were aligned to the 3'-end. Due to the readability reasons, only the 70 nt from the 3'-end are shown. The nucleotides are color-coded: adenosine – green, cytosine – blue, guanosine – yellow, uridine – red. The unexpected regular patterns of three consecutive nucleobases near 3'-end are shown in red frame.

Supplementary Figure 7

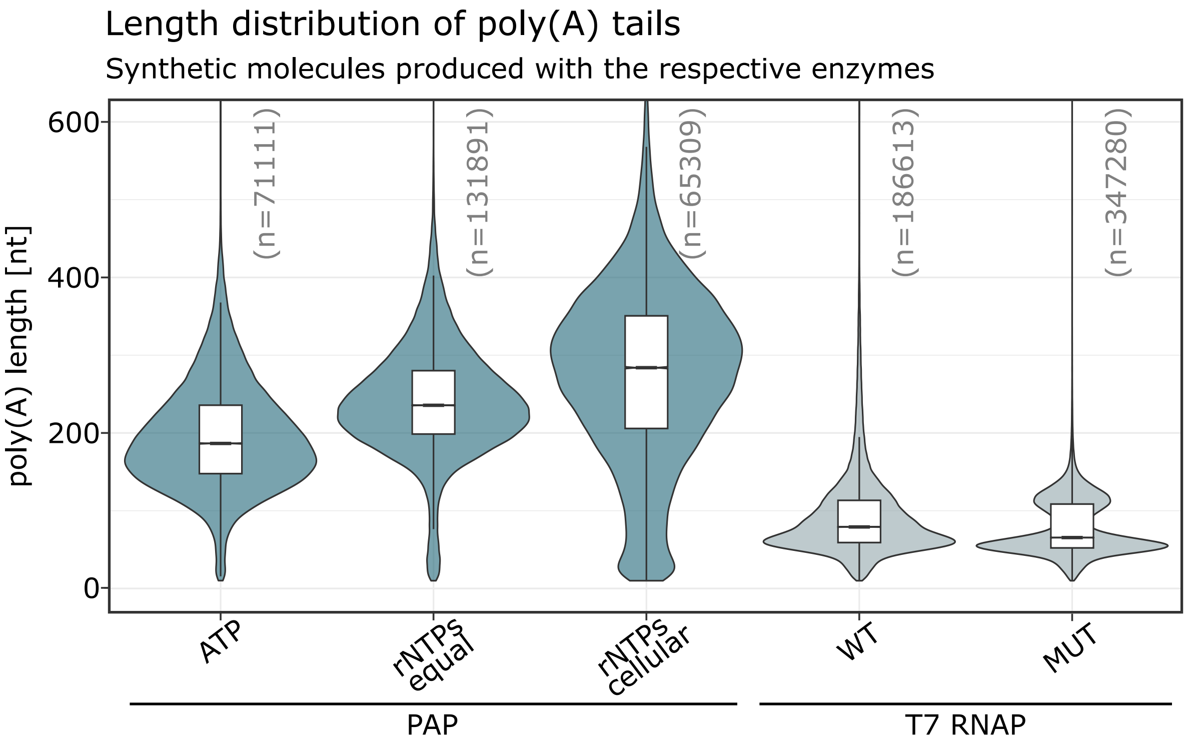

Supplementary Figure 7. Poly(A) tail length distribution in synthetic molecules.

PAP (teal) – synthetic molecules tailed with *E. coli* polyadenylate polymerase; T7 RNAP (light teal) – synthetic molecules produced with T7 polymerase (with PCR-templated tails); ATP – tailing reaction mix with ATP only; rNTPs equal – tailing reaction mix with equimolar concentration of rNTPs; rNTPs cellular – tailing reaction mix with concentrations of rNTPs mimicking physiological conditions; WT – reaction mix with wild-type T7 RNAP; MUT – reaction mix with the double mutant G47A + 884G T7 RNAP. Read counts are shown and median tail lengths are marked. In the boxplots, the horizontal lines represent the medians, while the lower and upper hinges correspond to the first and third quartiles (25th and 75th percentiles). The whiskers extend to the smallest and largest values within 1.5 times the interquartile range from the hinges.

Supplementary Figure 8

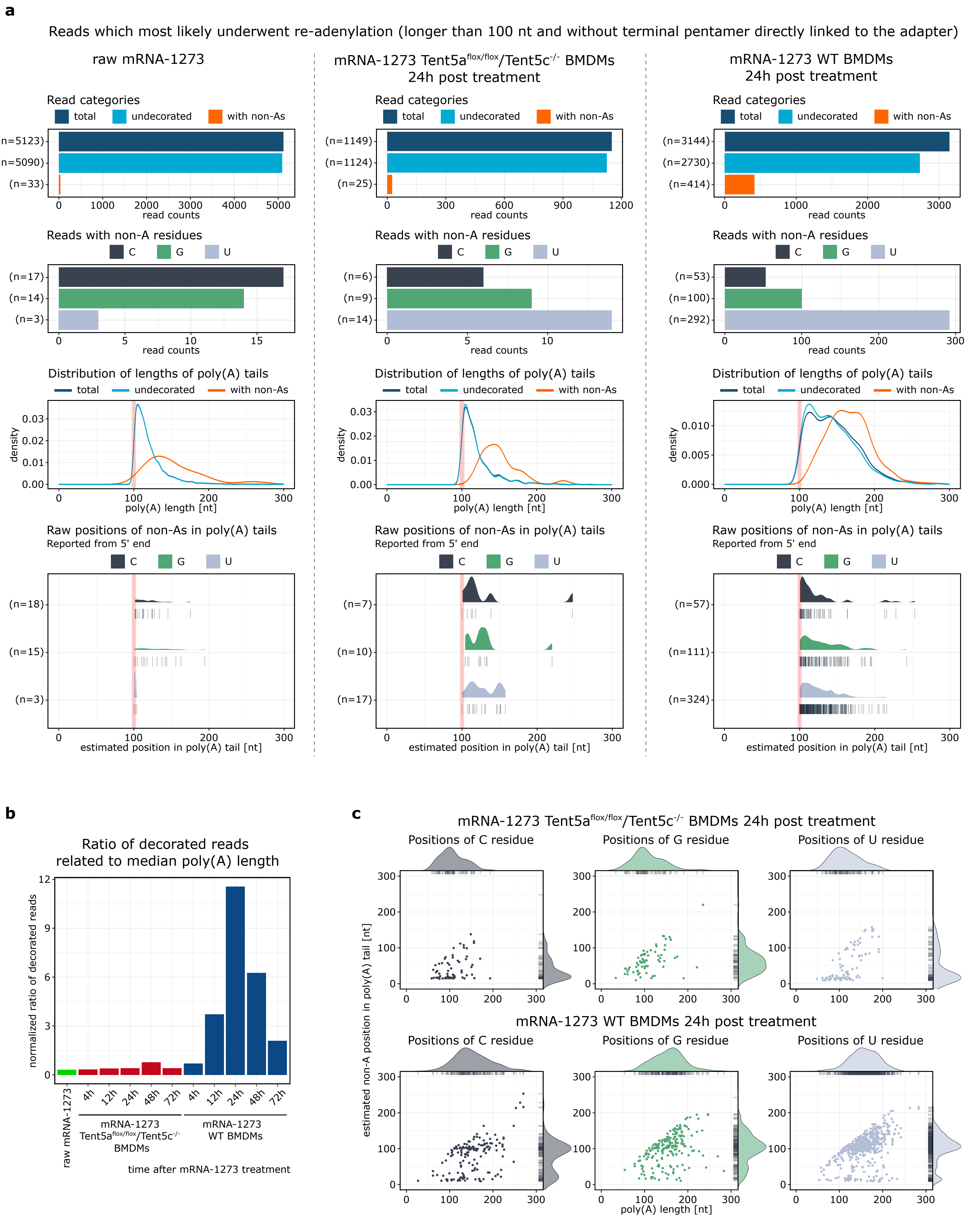

Supplementary Figure 8. Non-adenosine profiling in re-adenylated Moderna mRNA-1273 reads.

a, Detailed comparison of non-adenosine content in crude vaccine and vaccine reads isolated from BMDMs (Tent5a$^{Flox/Flox}$/Tent5c$^{-/-}$ or wild-type) 24h after administration. Reads which most likely underwent re-adenylation were selected: longer than 100 nt and without 3'-terminal mΨCmΨAG pentamer adjacent to the sequencing adapter. First row: frequency of read categories. Second row: frequency of tails with given non-adenosines. Third row: poly(A) tail length distribution of reads from either category. Fourth row: estimated positions of non-adenosines within poly(A) tails. In the third and fourth rows the expected position of 3'-terminal mΨCmΨAG pentamer (100 nt) is shown as red vertical line. In each panel, corresponding read counts (n) are provided. **b**, Ratio of decorated Moderna mRNA-1273 reads in relation to their median poly(A) length in crude vaccine and in vaccine reads isolated from BMDMs up to 72h after treatment. First, the ratio of decorated reads was computed, then multiplied by median tail length. **c**, Positions of non-adenosines within the poly(A) tails of Moderna mRNA-1273 reads 24h after administration to the BMDMs.

Supplementary Figure 9

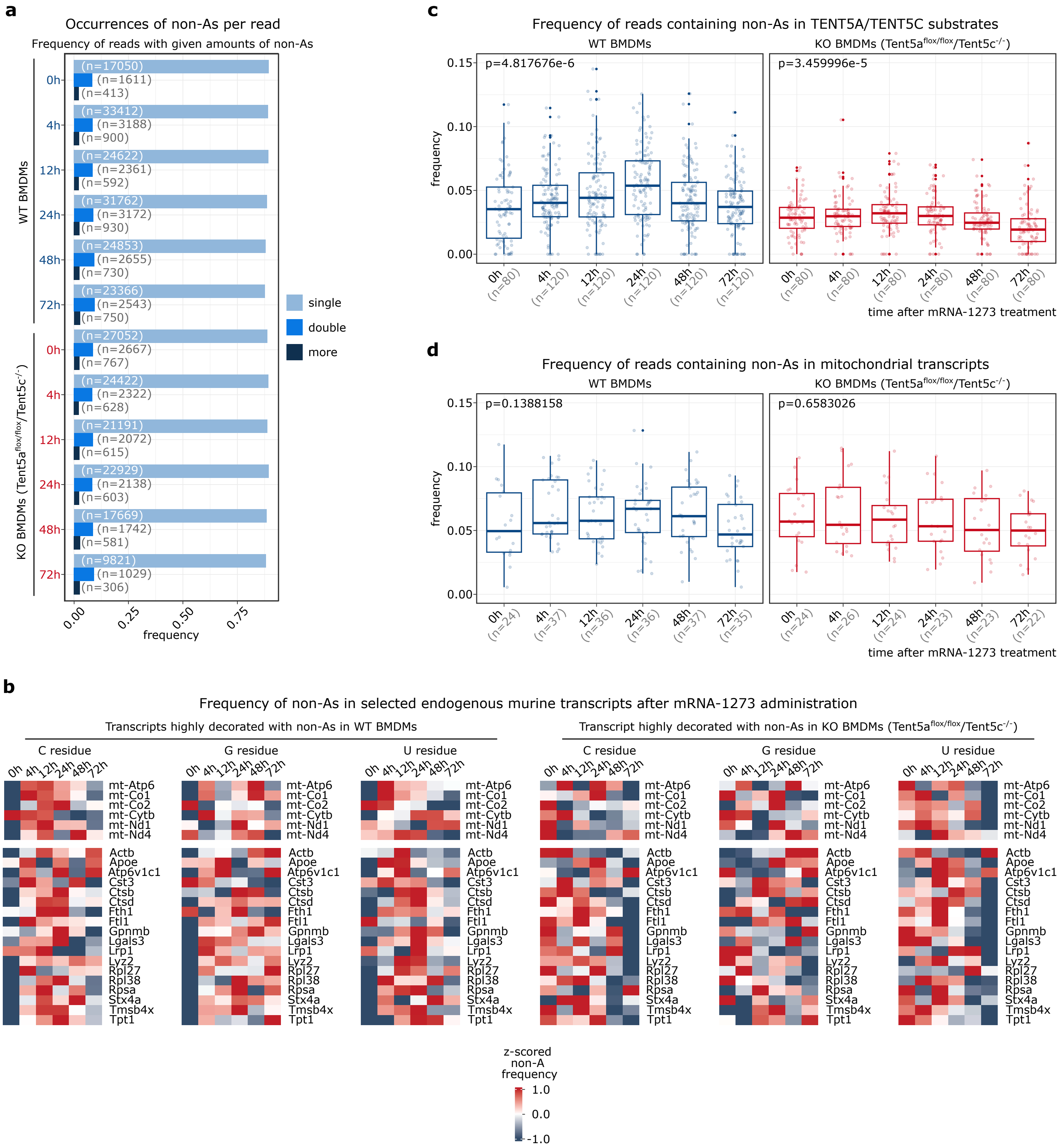

Supplementary Figure 9. Non-adenosine profiling in murine BMDMs.

a, Frequencies of decorated reads with given amount of non-adenosines in each BMDMs' phenotype/timepoint. **b**, Heatmaps showing changes of non-adenosine content in highly decorated genes up to 72h after Moderna mRNA-1273 administration. Genes with tails significantly enriched in non-adenosines were determined based on Fischer's exact test, two-tailed, with Benjamini-Hochberg correction, alpha = 0.05. **c**, Frequency of tails containing non-adenosines in TENT5A/C substrates for up to 72 h after mRNA-1273 treatment. P.values were calculated using Kruskal-Wallis rank sum test, alpha = 0.05. Corresponding observation counts (n) are provided. **d**, Frequency of tails containing non-adenosines in mitochondrially-encoded transcripts for up to 72 h after mRNA-1273 treatment. P.values were calculated using Kruskal-Wallis rank sum test, alpha = 0.05. Corresponding observation counts (n) are provided. For panels c, d containing boxplots, the horizontal lines represent the medians, while the lower and upper hinges correspond to the first and third quartiles (25th and 75th percentiles). The whiskers extend to the smallest and largest values within 1.5 times the interquartile range from the hinges.

Supplementary References

1. Brakel, J.-P. van. Robust Peak Detection Algorithm using z-Scores. (2014). at <https://stackoverflow.com/questions/22583391/peak-signal-detection-in-realtime-timeseries-data>

2. Kim, M. & Hargrove, L. J. Generating synthetic gait patterns based on benchmark datasets for controlling prosthetic legs. *J. NeuroEngineering Rehabil.* **20**, 115 (2023).

3. Catalbas, M. C. & Dobrisek, S. Dynamic speaker localization based on a novel lightweight R–CNN model. *Neural Comput. Appl.* **35**, 10589–10603 (2023).

4. Oeser, L., Samala, N., Hillemann, L., Rudolph, A. & Lienig, J. Minimizing the coincidence error in particle size spectrometers with digital signal processing techniques. *J. Aerosol Sci.* **165**, 106039 (2022).

5. Link, J., Perst, T., Stoeve, M. & Eskofier, B. M. Wearable Sensors for Activity Recognition in Ultimate Frisbee Using Convolutional Neural Networks and Transfer Learning. *Sensors* **22**, 2560 (2022).

6. Lima, B. M. R., Ramos, L. C. S., Oliveira, T. E. A. de, Fonseca, V. P. da & Petriu, E. M. Heart Rate Detection Using a Multimodal Tactile Sensor and a Z-score Based Peak Detection Algorithm. *CMBES Proc.* **42**, (2019).

7. Baskozos, G. *et al.* Comprehensive analysis of long noncoding RNA expression in dorsal root ganglion reveals cell-type specificity and dysregulation after nerve injury. *Pain* **160**, 463–485 (2019).

8. Perkins, P. & Heber, S. Identification of Ribosome Pause Sites Using a Z-Score Based Peak Detection Algorithm. in *2018 IEEE 8th International Conference on Computational Advances in Bio and Medical Sciences (ICCABS)* 1–6 (2018). doi:10.1109/ICCABS.2018.8541902.

9. Workman, R. E. *et al.* Nanopore native RNA sequencing of a human poly(A) transcriptome. *Nat. Methods* **16**, 1297–1305 (2019).

10. Krause, M. *et al.* tailfindr: alignment-free poly(A) length measurement for Oxford Nanopore RNA and DNA sequencing. *RNA* **25**, 1229–1241 (2019).

11. Niazi, A. M., Krause, M. & Valen, E. Transcript Isoform-Specific Estimation of Poly(A) Tail Length by Nanopore Sequencing of Native RNA. *Methods Mol. Biol.* **2284**, 543–567 (2021).
