# Supplementary material for: Direct profiling of non-adenosines in poly(A) tails of endogenous and therapeutic mRNAs with Ninetails
Source: Nat Commun. 2025 Mar 18;16:2664. doi: 10.1038/s41467-025-57787-6 (PMC11920217; doi:10.1038/s41467-025-57787-6)
Supplement: Supplementary file 2 — Description of Additional Supplementary Files [file 41467_2025_57787_MOESM2_ESM.pdf]

Description of Additional Supplementary Files

Supplementary Data 1 – Sample metadata, hardware and software specifics, CNN architecture and performance (benchmark).

Supplementary Data 2 – Summarized data used for orthogonal validation of Ninetails/Nanopore vs FLAMseq/PacBio based on datasets from publicly available repositories (Legnini et al. 2019 and Tavakoli et al. 2023). Source data for Figure 2.

Supplementary Data 3 – Summarized data used for comparison of non-adenosine incorporation frequency by various enzymes based on an in vitro synthesized molecules analyzed with Ninetails pipeline. Source data for Figure 3.

Supplementary Data 4 – Summarized data from moderna mRNA-1273-derived reads obtained as a result of crude mRNA-1273 and bone-marrow derived murine macrophages subjected to mRNA-1273 in various time points analyzed with Ninetails pipeline. Reads were classified based on pentamer (UCUAG) presence/absence with DTW algorithm described by Krawczyk et al. 2023. Source data for Figure 4.

Supplementary Data 5 – Summarized data from reads corresponding to murine endogenous transcripts bone-marrow derived murine macrophages subjected to mRNA-1273 in various time points analyzed with Ninetails pipeline. Source data for Figure 5.

Supplementary Data 6 – Summarized data from reads corresponding to murine endogenous transcripts from various cell types (bone-marrow derived murine macrophages, dendritic cells, B cells, T cells) analyzed with Ninetails pipeline. Source data for Figure 6.
