# Supplementary material for: Direct profiling of non-adenosines in poly(A) tails of endogenous and therapeutic mRNAs with Ninetails
Source: Nat Commun. 2025 Mar 18;16:2664. doi: 10.1038/s41467-025-57787-6 (PMC11920217; doi:10.1038/s41467-025-57787-6)
Supplement: Supplementary file 9 — Reporting Summary [file 41467_2025_57787_MOESM9_ESM.pdf]

nature portfolio

Corresponding author(s): Andrzej Dziembowski

Last updated by author(s): 29/09/24

Reporting Summary

Nature Portfolio wishes to improve the reproducibility of the work that we publish. This form provides structure for consistency and transparency in reporting. For further information on Nature Portfolio policies, see our Editorial Policies and the Editorial Policy Checklist.

Statistics

For all statistical analyses, confirm that the following items are present in the figure legend, table legend, main text, or Methods section.

n/a	Confirmed	
☐	☒	The exact sample size (n) for each experimental group/condition, given as a discrete number and unit of measurement
☐	☒	A statement on whether measurements were taken from distinct samples or whether the same sample was measured repeatedly
☐	☒	The statistical test(s) used AND whether they are one- or two-sided<br>*Only common tests should be described solely by name; describe more complex techniques in the Methods section.*
☐	☒	A description of all covariates tested
☐	☒	A description of any assumptions or corrections, such as tests of normality and adjustment for multiple comparisons
☐	☒	A full description of the statistical parameters including central tendency (e.g. means) or other basic estimates (e.g. regression coefficient) AND variation (e.g. standard deviation) or associated estimates of uncertainty (e.g. confidence intervals)
☐	☒	For null hypothesis testing, the test statistic (e.g. F, t, r) with confidence intervals, effect sizes, degrees of freedom and P value noted<br>*Give P values as exact values whenever suitable.*
☒	☐	For Bayesian analysis, information on the choice of priors and Markov chain Monte Carlo settings
☒	☐	For hierarchical and complex designs, identification of the appropriate level for tests and full reporting of outcomes
☐	☒	Estimates of effect sizes (e.g. Cohen's d, Pearson's r), indicating how they were calculated

Our web collection on statistics for biologists contains articles on many of the points above.

Software and code

Policy information about availability of computer code

Data collection	MinKNOW (Oxford Nanopore Technologies, multiple versions, updated frequently, from 18.12 to 22.10.10) was used for data collection during ONT RNA sequencing.<br><br>Guppy (Oxford Nanopore Technologies, versions 4.0.11, 4.4.1, 5.0.11, 6.0.0) was used for basecalling of RNA sequencing data.
Data analysis	Minimap 2.17, Nanopolish 0.13.2, Samtools 1.9, Guppy (versions 4.0.11, 4.4.1, 5.0.11, 6.0.0), Subread 2.0.1<br><br>R 4.1.2 with the following packages:<br>base, datasets, graphics, grDevices, grid, methods, stats, stats4, utils (all of them have the same version number as R)<br><br>Other R packages loaded via namespace: BiocGenerics 0.40.0, biomaRt 2.50.3 caret 6.0-93, ComplexHeatmap 2.10.0, dplyr 1.1.0, forcats 1.0.0, GenomeInfoDb 1.30.1, GenomicRanges 1.46.1, ggplot2 3.4.1, gprofiler2 0.2.1, Gviz 1.38.4, IRanges 2.28.0, keras 2.11.0, lattice 0.21-8, lubridate 1.9.2, nanotail 0.1.0, pROC 1.18.0, purrr 1.0.1, readr 2.1.4, reticulate 1.28, rhdf5 2.38.1, S4Vectors 0.32.4, stringr 1.5.0, tibble 3.1.8, tidyr 1.3.0, tidyverse 2.0.0, AnnotationDbi 1.56.2, AnnotationFilter 1.18.0, assertthat 0.2.1, backports 1.4.1, base64enc 0.1-3, Biobase 2.54.0, BiocFileCache 2.2.1, BiocIO 1.4.0, BiocParallel 1.28.3, Biostrings 2.62.0, biovizBase 1.42.0, bit 4.0.5, bit64 4.0.5, bitops 1.0-7, blob 1.2.4, BSgenome 1.62.0, cachem 1.0.8, checkmate 2.2.0, circlize 0.4.15, class 7.3-21, cli 3.6.0, clue 0.3-64, cluster 2.1.4, codetools 0.2-19, colorspace 2.1-0, compiler 4.1.2, crayon 1.5.2, curl 5.0.0, data.table 1.14.8, DBI 1.1.3, dbplyr 2.3.1, DelayedArray 0.20.0, deldir 1.0-6, dichromat 2.0-0.1, digest 0.6.31, doParallel 1.0.17, doSNOW 1.0.20, ensembldb 2.18.4, evaluate 0.20, fansi 1.0.4, farver 2.1.1, fastmap 1.1.1, filelock 1.0.2, foreach 1.5.2, foreign 0.8-84, Formula 1.2-5, future.apply 1.10.0,future 1.32.0, generics 0.1.3, GenomeInfoDbData 1.2.7, GenomicAlignments 1.30.0, GenomicFeatures 1.46.5, GetoptLong 1.0.5, GlobalOptions 0.1.2, globals 0.16.2, glue 1.6.2, gower 1.0.1, gridExtra 2.3, gtable 0.3.3,

hardhat 1.2.0, here 1.0.1, Hmisc 4.8-0, hms 1.1.3, htmlTable 2.4.1, htmltools 0.5.5, htmlwidgets 1.6.1, httr 1.4.5, interp 1.1-4, ipred 0.9-13, iterators 1.0.14, jpeg 0.1-10, jsonlite 1.8.4, KEGGREST 1.34.0, knitr 1.42, labeling 0.4.2, latticeExtra 0.6-30, lava 1.7.2.1, lazyeval 0.2.2, lifecycle 1.0.3, listenv 0.9.0, magrittr 2.0.3, MASS 7.3-58.2, Matrix 1.5-4, MatrixGenerics 1.6.0, matrixStats 0.63.0, memoise 2.0.1, ModelMetrics 1.2.2.2, munsell 0.5.0, nlme 3.1-162, nnet 7.3-18, parallel 4.1.2, parallelly 1.35.0, pillar 1.9.0, pkgconfig 2.0.3, plotly 4.10.1, plyr 1.8.8, png 0.1-8, prettyunits 1.1.1, prodlim 2019.11.13, progress 1.2.2, ProtGenerics 1.26.0, R6 2.5.1, rappdirs 0.3.3, RColorBrewer 1.1-3, Rcpp 1.0.10, RCurl 1.98-1.12, recipes 1.0.5, reshape2 1.4.4, restfulr 0.0.15, rhdf5filters 1.6.0, Rhdf5lib 1.16.0, rjson 0.2.21, rlang 1.1.1, rmarkdown 2.20, rpart 4.1.19, rprojroot 2.0.3, Rsamtools 2.10.0, RSQLite 2.3.0, rstudioapi 0.14, rtracklayer 1.54.0, scales 1.2.1, shape 1.4.6, snow 0.4-4, splines 4.1.2, stringi 1.7.12, SummarizedExperiment 1.24.0, survival 3.5-5, tensorflow 2.11.0, tfruns 1.5.1, tidyselect 1.2.0, timechange 0.2.0, timeDate 4022.108, tools 4.1.2, tzdb 0.3.0, utf8 1.2.3, VariantAnnotation 1.40.0, vctrs 0.5.2, viridisLite 0.4.1, vroom 1.6.1, whisker 0.4.1, withr 2.5.0, xfun 0.39, XML 3.99-0.14, xml2 1.3.4, XVector 0.34.0, yaml 2.3.7, zeallot 0.1.0, zlibbioc 1.40.0

python 3.8.15 with following packages:
absl-py 1.4.0, astunparse 1.6.3, cachetools 5.2.1, certifi 2022.12.7, charset-normalizer 3.0.1, flatbuffers 23.1.4, gast 0.4.0, google-auth 2.16.0, google-auth-oauthlib 0.4.6, google-pasta 0.2.0, grpcio 1.51.1, h5py 3.7.0, idna 3.4, importlib-metadata 6.0.0, keras 2.11.0, libclang15.0.6.1, markdown 3.4.1, markupsafe 2.1.1, numpy 1.24.1, oauthlib 3.2.2, opt-einsum 3.3.0, packaging 23.0, protobuf 3.19.6, pyasn1 0.4.8, pyasn1-modules 0.2.8, requests 2.28.2, requests-oauthlib 1.3.1, rsa 4.9, six 1.16.0, tensorboard 2.11.2, tensorboard-data-server 0.6.1, tensorboard-plugin-wit 1.8.1, tensorflow 2.11.0, tensorflow-estimator 2.11.0, tensorflow-io-gcs-filesystem 0.29.0, termcolor 2.2.0, typing-extensions 4.4.0, urllib3 1.26.14, werkzeug 2.2.2, wrapt 1.14.1, zipp 3.11.0

The code for subsequence Dynamic Time Warping is deposited at https://github.com/LRB-IIMCB/DTW_mRNA-1273.
The code for modified nanopolish-polya for detection of poly(A) terminal pentamer of mRNA-1273 is deposited at: https://github.com/LRB-IIMCB/nanopolish_mRNA-1273.

The Ninetails code is available at https://github.com/LRB-IIMCB/ninetails and https://doi.org/10.5281/zenodo.13309819.
Additional resources regarding Ninetails data processing including docker image are available at: https://github.com/LRB-IIMCB/ninetails_processing and https://zenodo.org/doi/10.5281/zenodo.13310034.

Full documentation for Ninetails is available at: https://github.com/LRB-IIMCB/ninetails/wiki

Software and their versions are also listed in Supplementary Data 1.

For manuscripts utilizing custom algorithms or software that are central to the research but not yet described in published literature, software must be made available to editors and reviewers. We strongly encourage code deposition in a community repository (e.g. GitHub). See the Nature Portfolio guidelines for submitting code & software for further information.

Data

Policy information about availability of data

All manuscripts must include a data availability statement. This statement should provide the following information, where applicable:
- Accession codes, unique identifiers, or web links for publicly available datasets
- A description of any restrictions on data availability
- For clinical datasets or third party data, please ensure that the statement adheres to our policy

Nanopore direct RNA sequences are deposited at the European Nucleotide Archive; accession number PRJEB53190 and PRJEB67899.

Data underlying figures are provided in Supplementary Datasets and are also publicly available from Zenodo along with additional raw data: https://zenodo.org/doi/10.5281/zenodo.13310034

PacBio FLAMseq data from Caenorhabditis elegans and HeLa cells (Legnini et al, Nat Meth, 2019) were downloaded from the NCBI GEO (accession number GSE126465).

ONT DRS data from HeLa cells (Tavakoli et al, Nat Commun, 2023) were downloaded from the NCBI SRA (accession number PRJNA777450).

Research involving human participants, their data, or biological material

Policy information about studies with human participants or human data. See also policy information about sex, gender (identity/presentation), and sexual orientation and race, ethnicity and racism.

Reporting on sex and gender	Not applicable.
Reporting on race, ethnicity, or other socially relevant groupings	Not applicable.
Population characteristics	Not applicable.
Recruitment	Not applicable.
Ethics oversight	Not applicable.

Note that full information on the approval of the study protocol must also be provided in the manuscript.

Field-specific reporting

Please select the one below that is the best fit for your research. If you are not sure, read the appropriate sections before making your selection.

☒ Life sciences ☐ Behavioural & social sciences ☐ Ecological, evolutionary & environmental sciences

For a reference copy of the document with all sections, see nature.com/documents/nr-reporting-summary-flat.pdf

Life sciences study design

All studies must disclose on these points even when the disclosure is negative.

Sample size	No statistical method was used to predetermine sample size. For in vivo animal experiments, we estimated the sample size based on previous studies, our pilot experiments.
Data exclusions	There was no formal criteria of sample exlusions. All attempts to repeat the experiments were successful.
Replication	Samples were produced in biological duplicates or triplicates with exception of T cells, which were repeated once. The exact number of biological replicates used for statistical anaysis is stated for every single experiment. As described above, samples with clear technical failures during processing or data collection were excluded from analyses.
Randomization	Mice of different genotypes were assigned with individual numerical tags in the database and they were used for the tissue collection and throughout subsequent processing as the only identifiers.
Blinding	Individual mice had an assigned numerical tag and mice with different genotypes were indistinguishable by the experimenter (except for Tent5a(-/-) mice which have distinguishable phenotype.

Reporting for specific materials, systems and methods

We require information from authors about some types of materials, experimental systems and methods used in many studies. Here, indicate whether each material, system or method listed is relevant to your study. If you are not sure if a list item applies to your research, read the appropriate section before selecting a response.

Materials & experimental systems

n/a	Involved in the study	
☒	☐	Antibodies
☐	☒	Eukaryotic cell lines
☒	☐	Palaeontology and archaeology
☐	☒	Animals and other organisms
☒	☐	Clinical data
☒	☐	Dual use research of concern
☒	☐	Plants

Methods

n/a	Involved in the study	
☒	☐	ChIP-seq
☒	☐	Flow cytometry
☒	☐	MRI-based neuroimaging

Eukaryotic cell lines

Policy information about cell lines and Sex and Gender in Research

Cell line source(s)	The primary Bone Marrow Derived Macrophages cell cultures were established from the bone marrow monocytes isolated from Tent5a(Flox/Flox)/Tent5c(-/-) and wild-type 12–25 weeks old mice of both sexes as described in Methods section of this manuscript.<br><br>The primary Bone Marrow Derived Dendritic Cells cultures were isolated from the femurs of 12-16 weeks old mice of both sexes as described in Methods section of this manuscript.<br><br>The primary splenic T cells were isolated from wild-type 12–16 weeks old mice of both sexes and stimulated with Dynabeads Mouse T-Activator CD3/CD28 Kit (Invitrogen). The details are described in Methods section of this manuscript.<br><br>The primary splenic B cells were isolated from wild-type 12–16 weeks old mice of both sexes and activated as described in Methods section of this manuscript.
Authentication	Cell lines were not authenticated.
Mycoplasma contamination	All cell lines tested negative for mycoplasm contamination by PCR.
Commonly misidentified lines (See ICLAC register)	No commonly misidentified lines were used in this study.

Animals and other research organisms

Policy information about studies involving animals; ARRIVE guidelines recommended for reporting animal research, and Sex and Gender in Research

Laboratory animals	All mice lines were generated by the CRISPR/Cas9-based method in the Genome Engineering Unit (https://crisprmice.eu/) at the International Institute of Molecular and Cell Biology in Warsaw, and described in details in Methods section of this manuscript.<br><br>Mice of both sexes were used for the experiments and sacrificed at age 12-25 weeks as detailed in Methods section.<br><br>All mice were bred at the animal house of Faculty of Biology, University of Warsaw. Mice were maintained in conventional conditions in open polypropylene cages filled with wood chip bedding (Rettenmaier) . Environment was enriched with nest material and paper tubes. Mice were fed at libutum with standard laboratory diet (Labofeed B, Morawski). Humidity in the rooms was kept at 55% ± 10%, temperature at 22oC ± 2oC, at least 15 air changes per hour, light regime set at 12h/12h (lights on from 6:00 to 18:00). Detail description of animals housing conditions are provided in the Methods section.<br><br>Caenorhabditis elegans animals (wild-type N2 Bristol), L4 were kept on NGM plates with E. coli HB101. 10 h after entering the L4 stage, they were used for RNA isolation. The worms were kept at the Laboratory of RNA Biology, International Institute of Molecular and Cell Biology in Warsaw.
Wild animals	No wild animals were used in the study.
Reporting on sex	Mice of both sexes were used at age 12-24 weeks. Worms of both sexes were used at age of 55h (10h after entering L4 stage).
Field-collected samples	No field collected samples were used in the study.
Ethics oversight	All procedures with animals were approved by the II Local Ethical Committee in Warsaw (approval numbers: WAW2/71/2021, WAW2/129/2021, WAW2/95/2022, WAW2/127/2022, WAW2/007/2023) with the requirements of the EU (Directive 2010/63/EU) and Polish (Act number 266/15.01.2015) legislation.

Note that full information on the approval of the study protocol must also be provided in the manuscript.

Plants

Seed stocks	No plants were used in this study.
Novel plant genotypes	No plants were used in this study.
Authentication	No plants were used in this study.
