# Supplementary material for: Direct profiling of non-adenosines in poly(A) tails of endogenous and therapeutic mRNAs with Ninetails
Source: Nat Commun. 2025 Mar 18;16:2664. doi: 10.1038/s41467-025-57787-6 (PMC11920217; doi:10.1038/s41467-025-57787-6)
Supplement: Supplementary file 10 — Transparent Peer Review file [file 41467_2025_57787_MOESM10_ESM.pdf]

nature portfolio

Peer Review File

Direct profiling of non-adenosines in poly(A) tails of endogenous and therapeutic mRNAs with Ninetails

Corresponding Author: Professor Andrzej Dziembowski

This file contains all reviewer reports in order by version, followed by all author rebuttals in order by version.

Version 0:

Reviewer comments:

Reviewer #1

(Remarks to the Author)
This study from the Dziembowski laboratory introduces "Ninetails," a neural network-based tool for accurately identifying and quantifying non-adenosines within poly(A) tails of endogenous and therapeutic mRNAs. The source data for this analysis are reads from direct sequencing using the Nanopore platform. Using this tool, the authors observe the widespread presence of non-adenosine modifications across various mRNA classes, cell types, and species, which impact mRNA fate. They further analyze the poly(A) tail metabolism of mRNA-based COVID vaccines developed by Moderna and Pfizer. They observe that the vaccines exhibit dynamic poly(A) tail compositions that change during their cellular lifespan. This discovery has important implications for mRNA therapeutic development, suggesting that tail composition can influence mRNA stability and efficacy. The authors provide evidence that Ninetails outperforms existing methodologies by providing a direct and bias-free approach to sequencing poly(A) tails, enhancing our understanding of mRNA regulation, and offering insights into designing more effective mRNA-based therapies.

This is a significant and timely study from one of the leading groups to apply Nanopore-based methods to studying RNA. I suggest only a few comments to the authors for their consideration.

While Nanopore is one of the most exciting and rapidly developing technologies and currently the only method for directly sequencing RNA (to my knowledge), it has two principal weaknesses. One is accuracy. While this has significantly improved with advances in algorithms and technology, the error rate is still higher than that of short-read sequencing. The other, perhaps more significant, is sequencing depth and coverage. Typically, Nanopore may not detect low-abundance transcripts, which may introduce bias into analysis. The authors could consider this when concluding that other methods, such as TAIL-seq, overestimate non-adenosine abundance compared to their method.

While I am not an expert on the statistical treatment of noisy data, the authors could expound more on their choice of z-score thresholding rather than other approaches, such as spline fitting. Is a choice of threshold that works well for one dataset always appropriate for another, especially in very noisy data where distinguishing between noise and signal becomes more nuanced? How are thresholds chosen? In that vein, does a model trained on 60 nt tails hold out well for much longer tails?

The authors used the Nanopolish polya module to determine the boundaries of the poly(A) tails but then commented on instances of misassignment of these. Have they considered applying TailFindR for poly(A) length estimates? TailFindR is optimized explicitly for poly(A) tail length measurement, while Nanopolish is a more general tool. Both methods differ in treating the raw Nanopore signal data, so a comparison, or at least a mention as to why Nanopolish is preferable, would be worthwhile.

Finally, I would note that I found the statement in lines 279-280 “...administration of mRNA-1273 in BMDMs leads to massive changes in gene expression…” to be problematic from an ethical point of view. The authors cite their own earlier study (that has not yet been peer-reviewed). Still, I urge them to carefully consider the public perception of such a strong statement that could be misinterpreted and misused, given that the present study examines the metabolism of COVID-19 vaccines, likely to generate public interest and discussion.

THERE ARE NO PRIVATE COMMENTS TO THE EDITOR.

Eugene Valkov, National Institutes of Health, U.S.A.

Reviewer #2

(Remarks to the Author)
In this paper, Gumińska et al. presented the Ninetails deep learning model to identify and quantify non-adenosines in mRNA polyA tails from nanopore sequencing signals. With Ninetails, the authors investigated polyA tailing during various biological processes, e.g. mRNA in vitro synthesis and mRNA vaccine production, as well as under diverse cellular contexts.

From a technical perspective, my major concern is how does Ninetails handle non-As that are close to each other, e.g. “UU” in Fig3e. In “training dataset preparation”, the authors described that “the classification model was trained using sequencing data corresponding to an in vitro-transcribed molecules equipped with a pure poly(A) tail or containing a single non-adenosine at fixed position”. Since, e.g. “UUs” were not included in the training data, I am wondering if they could be correctly detected. I think Ninetails trained with“single non-adenosine” should be good enough for addressing most real-world biological questions considering non-As are rare and sparse, but it would be great if the authors could explain how to handle non-As that are close to each other.

I also have some minor comments:

1. Are non-As randomly incorporated? Did the authors observe polyA tail “subtypes” in terms of their non-A positions?

2. For Fig1e and Extended Fig2c, I would suggest the authors to define positive and negative classes.

3. In Fig2g, are the dots represent genes, as in Fig6h?

4. In Fig3a, why the group “ATP” also contain non-As? Is this an experimental artifact, or related to the detection accuracy of Ninetails?

Version 1:

Reviewer comments:

Reviewer #1

(Remarks to the Author)
I thank the authors for addressing my concerns with thorough explanations and improving what was already an excellent manuscript.

Reviewer #2

(Remarks to the Author)
The authors have addressed all my questions, and I would recommend the paper for publication.

Open Access This Peer Review File is licensed under a Creative Commons Attribution 4.0 International License, which permits use, sharing, adaptation, distribution and reproduction in any medium or format, as long as you give appropriate credit to the original author(s) and the source, provide a link to the Creative Commons license, and indicate if changes were made.
In cases where reviewers are anonymous, credit should be given to 'Anonymous Referee' and the source.
The images or other third party material in this Peer Review File are included in the article's Creative Commons license, unless indicated otherwise in a credit line to the material. If material is not included in the article's Creative Commons license and your intended use is not permitted by statutory regulation or exceeds the permitted use, you will need to obtain permission directly from the copyright holder.
To view a copy of this license, visit https://creativecommons.org/licenses/by/4.0/

Gumińska at al., point-by-point responses to the reviewers' comments

We thank the reviewers for their overall positive opinion about our manuscript. We have addressed all the issues raised, significantly improving the manuscript. Therefore, we expect you will find this revised version suitable for publication.

Reviewer #1 :

This study from the Dziembowski laboratory introduces "Ninetails," a neural network-based tool for accurately identifying and quantifying non-adenosines within poly(A) tails of endogenous and therapeutic mRNAs. The source data for this analysis are reads from direct sequencing using the Nanopore platform. Using this tool, the authors observe the widespread presence of non-adenosine modifications across various mRNA classes, cell types, and species, which impact mRNA fate. They further analyze the poly(A) tail metabolism of mRNA-based COVID vaccines developed by Moderna and Pfizer. They observe that the vaccines exhibit dynamic poly(A) tail compositions that change during their cellular lifespan. This discovery has important implications for mRNA therapeutic development, suggesting that tail composition can influence mRNA stability and efficacy. The authors provide evidence that Ninetails outperforms existing methodologies by providing a direct and bias-free approach to sequencing poly(A) tails, enhancing our understanding of mRNA regulation, and offering insights into designing more effective mRNA-based therapies.

This is a significant and timely study from one of the leading groups to apply Nanopore-based methods to studying RNA.

We thank the reviewer for the positive opinion about our study.

I suggest only a few comments to the authors for their consideration.

1. While Nanopore is one of the most exciting and rapidly developing technologies and currently the only method for directly sequencing RNA (to my knowledge), it has two principal weaknesses. One is accuracy. While this has significantly improved with advances in algorithms and technology, the error rate is still higher than that of short-read sequencing. The other, perhaps more significant, is sequencing depth and coverage. Typically, Nanopore may not detect low-abundance transcripts, which may introduce bias into analysis. The authors could consider this when concluding

that other methods, such as TAIL-seq, overestimate non-adenosine abundance compared to their method.

We agree with the reviewer that low read coverage is a bottleneck of DRS and in some instances may potentially lead to some misinterpretations especially in case of low abundant transcripts. However, according to our findings, the addition of non-adenosine nucleotides for the majority of transcripts is a stochastic process. In most transcripts, the proportion of decorated tails does not exceed a few percent. Rarely expressed transcripts fit into this trend. Lowly expressed transcripts may be detected with increased depth of nanopore sequencing, like in our case BMDMs (Bone Marrow Derived Macrophages) for which collectively we have relatively high number of reads (total 11 822 862, including 9 379 910 fulfilling quality criteria) enabling conclusions even for low abundant mRNAs. Regarding accuracy, we do not find it a major bottleneck in this case, bearing in mind constant improvements of basecalling algorithms, and as with longer reads it does not prevent correct assignment of a read to transcript. In the case of poly(A) tails accuracy seems to be irrelevant, as poly(A) tails are not basecalled and require separate analysis algorithms (like nanopolish).

Although with TAIL-seq several important discoveries on poly(A) biology were possible, also regarding terminal poly(A) residues (which are missing in DRS), we are convinced that DRS with Ninetails outperforms other existing methodologies regarding quantifications of internal non-A residues in poly(A) tails.

2. While I am not an expert on the statistical treatment of noisy data, the authors could expound more on their choice of z-score thresholding rather than other approaches, such as spline fitting. Is a choice of threshold that works well for one dataset always appropriate for another, especially in very noisy data where distinguishing between noise and signal becomes more nuanced? How are thresholds chosen? In that vein, does a model trained on 60 nt tails hold out well for much longer tails?

In the manuscript, we opted to highlight aspects beyond algorithm selection or training to ensure interest among wide readership. However, we provide detailed explanation below and in Supplementary information.

During the initial phase of Ninetails development, we assessed multiple methods of handling noisy time series data, eventually implementing the modified van Brackel's (2014) thresholding algorithm. Our rendition of this thresholding algorithm has two key functions: (I) identifying

anomalies corresponding to non-adenosines with sufficient probability, (II) determining the positions of non-adenosines more accurately than the moves alone. The empirical variables were fine-tuned using the results of multiple independent sequencing runs. Our assessment encompassed diverse datasets, including both synthetic and biological samples. Notably, tailfindR also exploits numerous hardcoded values adjusted empirically and considered by the authors as universal (e.g., POLY_A_RNA_SPIKE_THRESHOLD). Our thresholding algorithm creates a vector of "pseudomoves", which is then contrasted with the corresponding moves produced by the Guppy basecaller. On this basis, fragments of the poly(A) tail potentially containing non-adenosine are selected for subsequent steps. It should be noted that the further input to the neural network is extracted from the signal, not from "pseudomoves" (as shown on Extended data figure 1, discussed in Supplementary information, and shown on Supplementary figure 3). We have refined this part of the manuscript to avoid confusion.

The raw signal is winsorized and interpolated (which is a common practice in nanopore data processing, especially for visualization purposes). We chose this approach as most suitable after testing numerous signal denoising and compression methods (see Supplementary information, especially Supplementary figure 2). We applied this to improve the computational efficiency of Ninetails so it can be used not only on computational servers but also on desktop computers.

We understand reviewer's concerns regarding the training set made of synthetic RNA molecules with fixed poly(A) tails of 60 nucleotides. This, however, does not affect the accuracy of the model or the quality of the prediction. That is because the input to the neural network is a set of pre-filtered fragments of tail signals, not the entire poly(A) tails. These fragments have a fixed length covering 100 data points (the sequencer records observations in a fixed intervals and the molecule velocity is also considered constant). In the manuscript, we discuss the results from murine BMDMs, including transcripts with the longest tails and mitochondrial transcripts possessing short poly(A) tails (Figure 5).

3. The authors used the Nanopolish polya module to determine the boundaries of the poly(A) tails but then commented on instances of misassignment of these. Have they considered applying TailFindR for poly(A) length estimates? TailFindR is optimized explicitly for poly(A) tail length measurement, while Nanopolish is a more general tool. Both methods differ in treating the raw Nanopore signal data, so a comparison, or at least a mention as to why Nanopolish is preferable, would be worthwhile.

We thank reviewer for this comment. Over the past five years, we have extensively used nanopore direct RNA sequencing combined with the nanopolish polya to study polyadenylation of RNAs from various organisms, consistently yielding convincing and reliable results. During that time we benchmarked available algorithms, including tailfindR, on various datasets, consistently obtaining higher quality data with nanopolish polya/ In general, both programs provided compatible predictions regarding coordinates and estimated tail length. Nevertheless, tailfindR misidentified the tail region in a notable fraction of signals (up to 10% in certain sequencing runs), while nanopolish polya consistently provided accurate results, validated through examination of raw signals alongside predicted coordinates.

A significant concern with tailfindR is its tendency to erroneously estimate tails containing potential non-adenosines in close proximity to the tail boundaries. It often recognizes only the part of the tail that precedes or follows the non-adenosine (see Supplementary Information). Additionally, tailfindR's computational efficiency is inferior to nanopolish polya. We prioritized efficiency in Ninetails, thus selected tail detection software optimized for large datasets. Also, unlike tailfindR, nanopolish polya incorporates signal quality assessment, enabling exclusion of signals deemed too unreliable for classification. Consequently, we consider nanopolish polya a more dependable tool, hence adopting it as the default counterpart for Ninetails.

Furthermore, our aim was seamless integration of Ninetails into our internal pipelines and compatibility with our nanopore data analysis package, NanoTail (https://github.com/LRB-IIMCB/nanotail).

Nevertheless, we decided to accommodate the needs raised by the reviewer. The latest version of Ninetails available on Github (v.1.0.2) (https://github.com/LRB-IIMCB/ninetails) can also use the coordinates reported by tailfindR. The instructions are provided in the built-in manual and on Ninetails Wiki (https://github.com/LRB-IIMCB/ninetails/wiki). However, due to the serious concerns, we strongly advocate for employing nanopolish polya outputs over tailfindR to mitigate potential bias.

We have edited our manuscript and provided additional remarks and graphics in Supplementary Information to address this topic more thoroughly.

4. Finally, I would note that I found the statement in lines 279-280 "...administration of mRNA-1273 in BMDMs leads to massive changes in gene expression…"

to be problematic from an ethical point of view. The authors cite their own earlier study (that has not yet been peer-reviewed). Still, I urge them to carefully consider the public perception of such a strong statement that could be misinterpreted and misused, given that the present study examines the metabolism of COVID-19 vaccines, likely to generate public interest and discussion.

We thank the reviewer for spotting this out, indeed our statement was quite bold without the proper context. This part of the manuscript was rephrased.

Reviewer #2:

In this paper, Gumińska et al. presented the Ninetails deep learning model to identify and quantify non-adenosines in mRNA polyA tails from nanopore sequencing signals. With Ninetails, the authors investigated polyA tailing during various biological processes, e.g. mRNA in vitro synthesis and mRNA vaccine production, as well as under diverse cellular contexts.

1. From a technical perspective, my major concern is how does Ninetails handle non-As that are close to each other, e.g. “UU” in Fig3e. In “training dataset preparation”, the authors described that “the classification model was trained using sequencing data corresponding to an *in vitro*-transcribed molecules equipped with a pure poly(A) tail or containing a single non-adenosine at fixed position”. Since, e.g. “UUs” were not included in the training data, I am wondering if they could be correctly detected. I think Ninetails trained with “single non-adenosine” should be good enough for addressing most real-world biological questions considering non-As are rare and sparse, but it would be great if the authors could explain how to handle non-As that are close to each other.

We thank the reviewer for this interesting comment. Indeed, multiple non-adenosines per tail are rarely observed (Figure. 2c in the revised manuscript), and, as the reviewer aptly noted, they occurence in a row is scarce. Moreover, a well-known limitation of nanopore sequencing is the accuracy of low-complexity regions, also known as the homopolymer effect. For this reason, the Guppy basecaller from Oxford Nanopore does not offer the ability to accurately basecall and delimit poly(A) tails. The poly(A) tail delimitation and measurement is done instead with dedicated tools developed by the community: the Nanopolish polya and tailfindR. Given both factors (the overwhelming prevalence of single non-adenosines over

other potential arrangements) and the technical limitations of the nanopore itself, we decided to train our basic model on single nucleotide contexts. Our analysis in this article shows that the quality of these predictions allows us to formulate answers to most biological questions.

In some cases, Ninetails can report non-adenosines located close to each other as separate instances (example is shown on Figure 2d in the revised manuscript). Nevertheless, this is not the rule and depends very much on the quality of the signal. Finally, Ninetails is not limited to its built-in default model. The program includes several features that allow the user to prepare their own training and validation sets to create their own model. Also, one that considers additional nucleotide contexts. We also consider including such models in the future versions of Ninetails.

I also have some minor comments:

1. Are non-As randomly incorporated? Did the authors observe polyA tail “subtypes” in terms of their non-A positions?

In the vast majority of cases we observe a random distribution of non-adenosines. This phenomenon is illustrated on Figure 4d and Extended data figure 5 in the revised manuscript. The only exceptions we observed are endogenous transcripts with semi-templated tails (e.g., *Tmed9*), where we observe significantly increased concentration of non-adenosines in the templated part of poly(A), and composite tail of Moderna vaccine.

2. For Fig1e and Extended Fig2c, I would suggest the authors to define positive and negative classes.

We apologize for the clutter which might arise from lack of the proper description. To enhance comprehension we opt to represent the confusion matrix, typically organized in 2x2 format, graphically. Through graphical depiction, with bar size proportional to the number of observations associated with each outcome, we aim to facilitate interpretation of results. In our graphical representations, conventional terms are used: ‘positives’ refer to true positives and false positives, while ‘negatives’ refer to true negatives and false negatives, as stated in figure legend. The exact confusion matrix (source for the graphics) can be found in Supplementary table 1. Captions have been clarified.

3. In Fig2g, are the dots represent genes, as in Fig6h?

Yes, in both figures the dots represent genes. Captions have been clarified.

4. In Fig3a, why the group "ATP" also contain non-As? Is this an experimental artifact, or related to the detection accuracy of Ninetails?

It is an artifact of the experimental procedure. RNAs used in this experiment were synthesized in the 2-step reaction, with transcript body obtained by in vitro transcription (IVT) from the DNA template, followed by the polyadenylation reaction. Although IVT RNA went through clean-up procedure, traces of rNTPs co-purified with the RNA and were incorporated (at low rate) by PAP in the polyadenylation reaction. Examination of raw nanopore signals confirmed that we are indeed dealing with decorated tails, and Ninetails is reporting them correctly. Nevertheless, this does not hinder the proper interpretation of obtained results.
